# Sequencing the extrachromosomal circular mobilome reveals retrotransposon activity in plants

**DOI:** 10.1371/journal.pgen.1006630

**Published:** 2017-02-17

**Authors:** Sophie Lanciano, Marie-Christine Carpentier, Christel Llauro, Edouard Jobet, Dagmara Robakowska-Hyzorek, Eric Lasserre, Alain Ghesquière, Olivier Panaud, Marie Mirouze

**Affiliations:** 1 Institut de Recherche pour le Développement (IRD), UMR232 DIADE, 911 Avenue Agropolis, Montpellier, France; 2 University of Perpignan, Laboratory of Plant Genome and Development, 58 Avenue Paul Alduy, Perpignan, France; 3 Centre National de la Recherche Scientifique (CNRS), Laboratory of Plant Genome and Development, 58 Avenue Paul Alduy, Perpignan, France; University of Utah School of Medicine, UNITED STATES

## Abstract

Retrotransposons are mobile genetic elements abundant in plant and animal genomes. While efficiently silenced by the epigenetic machinery, they can be reactivated upon stress or during development. Their level of transcription not reflecting their transposition ability, it is thus difficult to evaluate their contribution to the active mobilome. Here we applied a simple methodology based on the high throughput sequencing of extrachromosomal circular DNA (eccDNA) forms of active retrotransposons to characterize the repertoire of mobile retrotransposons in plants. This method successfully identified known active retrotransposons in both Arabidopsis and rice material where the epigenome is destabilized. When applying mobilome-seq to developmental stages in wild type rice, we identified *PopRice* as a highly active retrotransposon producing eccDNA forms in the wild type endosperm. The mobilome-seq strategy opens new routes for the characterization of a yet unexplored fraction of plant genomes.

## Author summary

Long time considered as « junk DNA », the evolutive force of transposable elements (TEs) is now well established and TEs contribute strongly to eukaryote genome plasticity. However, it is difficult to fully characterize the mobile part of a genome, or active mobilome, and tracking TE activity remains challenging. We therefore propose to use the detection of extrachromosomal circular DNA as a diagnostic for plant TE activity. Our mobilome-seq technique allowed to identify a new active retrotransposon in wild type rice seeds, and will represent a powerful strategy in characterizing the somatic activity of TEs to evaluate their impact on genome stability and to better understand their adaptive capacity in multicellular eukaryotes.

## Introduction

Transposable elements (TEs) are major players in the evolution of animal and plant genomes [1–3]. The observation of both a complex epigenetic repression of TE expression and a large compartment occupied by TE copies in most sequenced eukaryotic genomes reflects a fine-tuned interaction between TEs and their host genomes [1–4]. TE proliferation in genomes leads to increased genomic diversity through mutations, genomic rearrangements like translocations or inversions [2], and epigenetic modifications [5]. This proliferation can also have a regulatory effect on gene expression that has been proposed to potentially result in adaptive traits [1,6,7].

According to their mode of transposition, TEs are organized into two main classes: retrotransposons (RTs) and DNA transposons (DNA-TEs). RTs multiply using a **«** copy and paste **»** strategy mediated by an RNA-intermediate, whereas DNA-TEs use a « cut and paste » mechanism [8]. During their life cycle TEs thus can exist as integrated DNA, mRNA and extrachromosomal linear DNA (S1 Fig). The extrachromosomal linear form, typical of actively proliferating TEs, can be detected by the host and may be circularized by DNA repair processes. The non-homologous end-joining mechanism and/or homologous recombination between flanking repeat sequences have been proposed to promote the circularization of extrachromosomal DNA into extrachromosomal circular DNA (eccDNA) [9–12]. There is no evidence that these eccDNAs can be re-integrated into the plant genome. Thus the formation of eccDNAs by the host could be a mechanism to limit the number of new insertions of active TEs in the genome (S1 Fig). Different types of active TEs have been detected as eccDNAs in plants such as *Tto1* [13], *Mu* [14] and *Ac/Ds* [15], however no genome-wide analysis of these forms has been performed yet. The mobilome consists of all mobile genetic elements in a cell that can be plasmids in prokaryotes or TEs in eukaryotes [16]. We will hereafter refer to the extrachromosomal forms of TEs as the reverse-transcribed mobilome.

Multiple approaches have been used to identify actively proliferating TEs at different steps of their life-cycle: (1) positional cloning of genes altered by a TE insertion (for example in rice the *hAT* DNA-TE [17] or the Long Terminal Repeat RT (LTR-RT) *Houba* [18], (2) search for TE-insertion polymorphisms using transposon display on candidate TEs (for example rice *mPing* and *Pong* [19], (3) transcription studies on candidates TEs using primers targeting conserved domains, for example rice LTR-RT *Tos17* [20] or through genome-wide transcriptomic analyses, for example the LTR-RT *Lullaby* in rice calli [21]. Today the most advanced technique to identify actively proliferating TEs in species where the genome sequence is available consists of whole-genome resequencing and detection of TE-associated polymorphisms using paired-end mapping [22–24].

The techniques listed above have important limitations. The analysis of transcripts by RNA-seq allows the description of transcriptionally active retrotransposons but does not take into account their capacity to produce proteins. As transcription is the first step in a retrotransposon life cycle, most copies do not go further this point, either because of post-transcriptional gene silencing activities or because they have accumulated mutations that prevent the translation of mature proteins, although some TEs with non functional proteins might parasite other TEs [25,26]. The analysis of neo-insertions through genome resequencing is very powerful to reduce the complexity of transcriptionally active TEs to the ones that effectively produce new insertions. This approach detects breakpoints between neo-insertions and a reference genome and thus requires a high sequencing coverage more adapted to small genomes. Furthermore only fixed, transgenerational neo-insertions can be detected with high accuracy. Finally, despite the numerous pipelines developed to characterize these neo-insertions [27,28], only insertions into non repetitive regions of the genome can be accurately detected, leaving a large part of the structural variations caused by TEs undetectable. Alternative approaches initially developed in mammals, such as retrotransposon-capture sequencing, consist in the enrichment and identification of the flanking sequences of a particular retrotransposon [28–30], but these techniques require prior knowledge of the active TE families in the species of interest. We therefore endeavor to develop a genome-wide strategy that could efficiently track potentially active TEs without full genome resequencing.

We sought to take advantage of the presence of circular forms of active TEs in the eccDNA compartment to identify active TEs in plants. Extrachromosomal DNA circles were identified decades ago in Drosophila [9,31] and observed by electron microscopy in *Vigna radiata* [32] and by two-dimensional gel-electrophoresis in carcinogen-treated cells [33] and in plants [34]. These eccDNAs can be formed by homologous recombination between adjacent repeats such as amplified genes [35], tandem repeats (satellite, telomeric, centromeric and ribosomal repeats) [34,36] or they can result from the linear extrachromosomal forms of active TEs [37]. These eccDNAs are ubiquitous elements and heterogeneous populations of eccDNAs seem to be present in all eukaryotic organisms [38]. Recently, sequencing of eccDNAs was experimented in mouse cells where microDNAs originating from chromosomal micro-deletions at specific gene loci [39,40] were identified. Numerous eccDNAs were detected in yeast cells [41,42], although no new active TE could be identified. Therefore, the abundance and identity of eccDNAs specifically resulting from the circularization of extrachromosomal TE DNA in multicellular organisms is not well documented. Here, we used the identification of TE eccDNA as a tool to investigate TE activation in plants and developed a dedicated computational pipeline to address this question.

We analyzed the active mobilomes from the two plant species *Arabidopsis thaliana* and *Oryza sativa*. As a proof of concept, we selected plant material where active TEs had previously been identified: a partially hypomethylated line for *A*. *thaliana* [43] and a callus tissue for *O*. *sativa*. Our mobilome-seq analyses clearly identified the two known active LTR-RTs *EVD* [44] and *Tos17* [45], in *A*. *thaliana and O*. *sativa* samples respectively, in their eccDNA forms. To investigate novel TE activity we applied mobilome-seq to wild type rice seeds and identified *PopRice* LTR-RTs as producing large amounts of eccDNAs specifically in the endosperm tissue. We propose that the mobilome-seq strategy could help identifying mobile TEs in different species to better understand the impact of the active mobilome on the host genome.

## Results

### Enrichment and sequencing of eccDNAs

**Fig 1:**
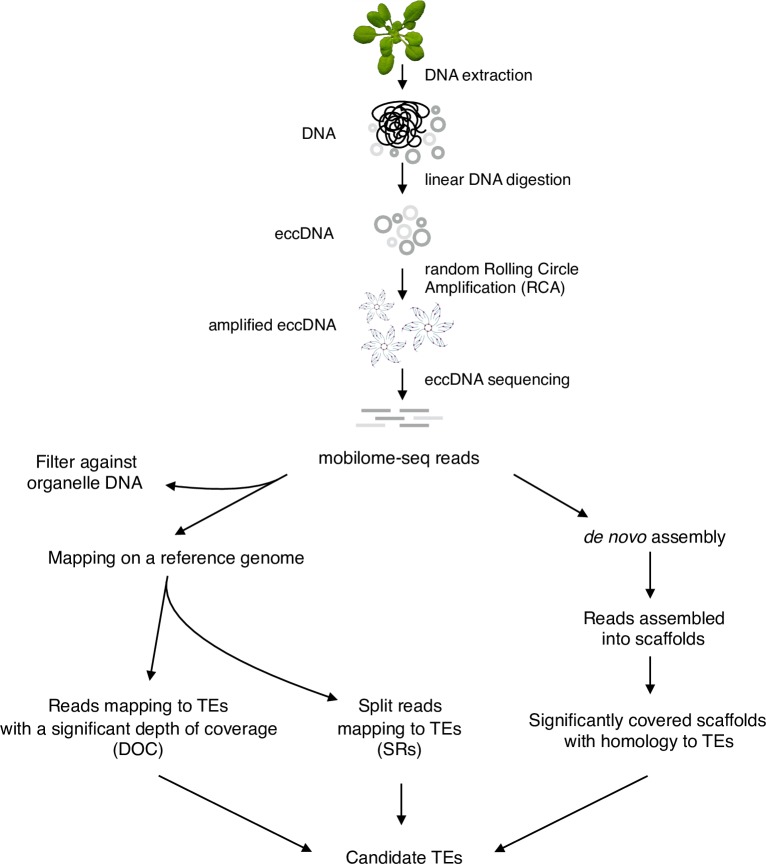
The mobilome-seq approach in plants. A schematic view of the main steps involved in the selection and amplification of the extrachromosomal circular molecules in plants. After DNA extraction, linear DNA molecules are digested and circular molecules are randomly amplified using rolling circle amplification. This DNA material is used for high-throughput sequencing. Mobilome-seq data analysis consists in characterizing the depth of coverage (DOC) of mapped reads and the presence of split reads (SRs) at TE loci and the detection of *de novo* assembled scaffolds corresponding to these TEs.

**Fig 2:**
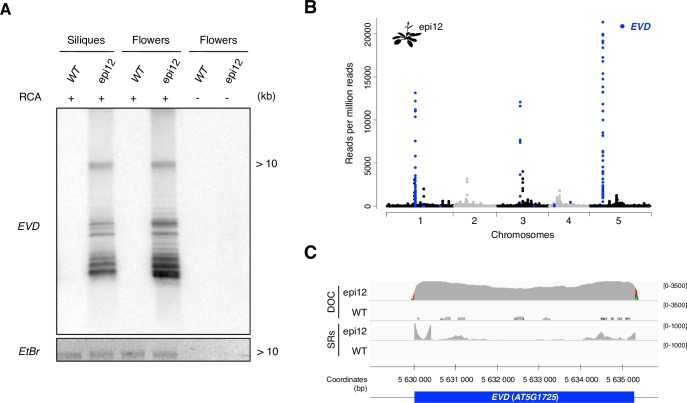
Mobilome-seq detection of *EVD*, a known active retrotransposon in Arabidopsis. (**A**) Southern blot experiment using *Hind*III-digested eccDNAs amplified from *A*. *thaliana* WT and epi12 flowers and siliques and detected with a probe specific for the *EVD* retrotransposon active in the line epi12. RCA: rolling circle amplification. The ethidium bromide (*EtBr*) gel picture is shown as a loading control. (**B**) Abundance of reads mapping at TE-annotated loci in the *A*. *thaliana* epi12 line mobilome-seq library. Each dot represents the normalized coverage per million mapped reads per all TE-containing 100bp windows obtained after aligning the sequenced reads on the *A*. *thaliana* reference genome. Blue dots indicate the windows corresponding to annotated *EVD* genomic loci. (**C**) Detail of the depth of coverage of total mapped reads (DOC) and split reads (SR) abundance of the *A*. *thaliana* epi12 and WT mobilome-seq data at the *EVD* locus on chromosome 5 (*blue bar*). Grey peaks: read abundance (not normalized), DOC: depth of coverage for all aligned reads, SRs: split reads, WT: wild type siliques, epi12: epi12 siliques. Maximum coverage is indicated on the right. Colors indicate the presence of SNPs.

In order to isolate and to sequence eccDNAs, total DNA was first extracted from plant tissues (Fig 1). Linear genomic DNA was digested with an exonuclease and the remaining eccDNA molecules were then amplified by rolling circle amplification (RCA) using random primers. This method does therefore not require any *a priori* knowledge on TEs for a given sample. We first performed this experiment on samples from *A*. *thaliana* Columbia wild type plants as a negative control (Col WT) and on an epigenetic recombinant inbred line (epiRIL12 hereafter called epi12) where an hypomethylated retrotransposon (*EVD*/*ATCOPIA93*) was detected as actively proliferating [44], as a positive control. Southern blot validation assays using an *EVD* specific probe were performed to analyze the enrichment of eccDNAs before and after the RCA step (Fig 2A). A signal corresponding to digested *EVD* eccDNAs was detected in samples from siliques and flowers from epi12 plants after RCA, but not in samples from WT plants. No signal could be detected in the absence of RCA indicating that most genomic DNA had been degraded after the exonuclease treatment. We used this material for high throughput sequencing.

### Detection of *EVD* by mobilome-seq in *A*. *thaliana* hypomethylated plants

We performed mobilome-seq on Col WT and epi12 siliques samples as shown in Fig 1. After mapping the reads on the reference genome of *A*. *thaliana* we detected peaks of high coverage in both WT and epi12 mobilome-seq libraries (S2 Fig) corresponding to ribosomal DNA (rDNA) loci that are known to produce eccDNAs [34]. All peaks of high coverage corresponding to TEs in both WT and epi12 are listed in S5 Table. In particular, peaks corresponding to *EVD* were specifically detected in epi12 (Fig 2B, S3A Fig and S5 Table). *EVD* is a 5,3 kilobases (kb)-long LTR-RT present in two full-length copies in the genome of *A*. *thaliana* ecotype *Columbia*. *EVD* is transcribed and mobilized in *met1*-derived epiRILs [44] and produces eccDNA copies [46]. Due to the repetitive nature of TEs, reads corresponding to *EVD* eccDNA can map against full-length and also truncated copies present in the genome explaining why all regions corresponding to *EVD* are more or less covered. Nevertheless, the two full-length copies (on chromosomes 1 and 5) are the most significantly covered with a p-value < 10^−8^ (S3B and S3C Fig). The *EVD* locus on chromosome 5 is highly covered in the epi12 mobilome-seq library compared to the WT library, with a depth of coverage (DOC) of 3500X versus 1X, respectively (Fig 2C). To further identify the presence of reads corresponding to eccDNA junctions, we specifically detected split reads (SRs) as paired-reads that are not correctly mapped onto the reference genome (*see* Methods). We could detect a high number of SRs at both 5’ and 3’ ends of *EVD* in the epi12 mobilome-seq data compared to WT (Fig 2C and S4 Fig) suggesting the presence of reads spanning the circular junction. A closer examination of some of these reads revealed that they indeed correspond to 2LTR junctions (S5 Fig). While 142 TEs were detected as overexpressed in epi12 at the transcriptional level [47], the mobilome-seq data suggest that only *EVD* produce circular copies (S6 Fig).

### *Tos17* is highly enriched in the *O*. *sativa* callus tissue mobilome-seq library

**Fig 3:**
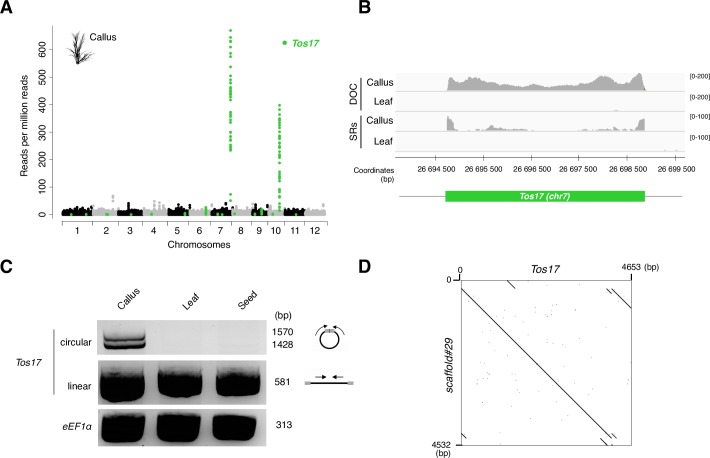
Mobilome-seq detection of *Tos17*, a known active retrotransposon in rice callus. (**A**) Abundance of reads mapping at TE-annotated loci in the *O*. *sativa* WT callus mobilome-seq library. Each dot represents the normalized coverage per million mapped reads per all TE-containing 100bp windows obtained after aligning the sequenced reads on the *O*. *sativa* reference genome. Green dots indicate the windows corresponding to annotated *Tos17* genomic loci. (**B**) Detail of the depth of coverage of total mapped reads (DOC) and split reads (SRs) abundance of the *O*. *sativa* WT callus and leaf mobilome-seq library at the *Tos17* locus on chromosome 7 (*green bar*). Legend as in Fig 2C. (**C**) Detection of circular forms of *Tos17* using inverse PCR with primers localization depicted on the right (*black bar*: *Tos17* element, *arrows*: PCR primers, *grey boxes*: LTRs). Upper gel: PCR amplification of *Tos17* circles, middle gel: control PCR for *Tos17* detection, lower gel: PCR using *eEF1α* primers as loading control. (**D**) Dotter alignment of the scaffold #29 obtained after *de novo* assembly of callus mobilome-seq library and *Tos17*.

We then analyzed mobilome-seq libraries from *O*. *sativa* ssp *japonica* cv *Nipponbare*, a species with a larger genome (400Mb) than *A*. *thaliana* (135Mb) and a three times bigger proportion of TEs (45% in *O*. *sativa* against 15% in *A*. *thaliana*), using both leaf material and callus tissue. TEs with high coverage in *O*. *sativa* mobilome-seq libraries are listed in S5 Table. More specifically, peaks corresponding to the *Tos17* family were detected in the mobilome-seq libraries of callus tissue but not in leaves (Fig 3A and 3B, S7 Fig). *Tos17* is a 4,1 kb-long LTR-RT present in two copies in the *O*. *sativa* genome (on chromosomes 7 and 10), the copy on chromosome 7 being active in calli [13]. The DOC analysis indicated a clear enrichment (DOC = 200X) at the *Tos17* locus on chromosome 7 in the callus mobilome-seq library compared to the leaf mobilome-seq library (<1X) (Fig 3B and S7B Fig). SRs were detected on both ends of *Tos17* suggesting the presence of reads spanning the junction. The presence of *Tos17* eccDNAs was confirmed by an inverse PCR assay (Fig 3C) and a closer inspection of SRs identified reads spanning the 2LTR-circle junction (S8A Fig). Moreover we have also analyzed the coverage of *Lullaby*, a LTR-RT active in calli [21]. A low coverage from 10X to 12X was detected in the callus mobilome-seq library and the presence of reads spanning junction of *Lullaby* eccDNAs was confirmed (S8B Fig). Altogether, these results show that known active LTR-RTs could be detected using the mobilome-seq approach, suggesting that this technique can be used to identify new active TEs in plants.

### Identification of a new active LTR-RT in wild type rice

**Fig 4:**
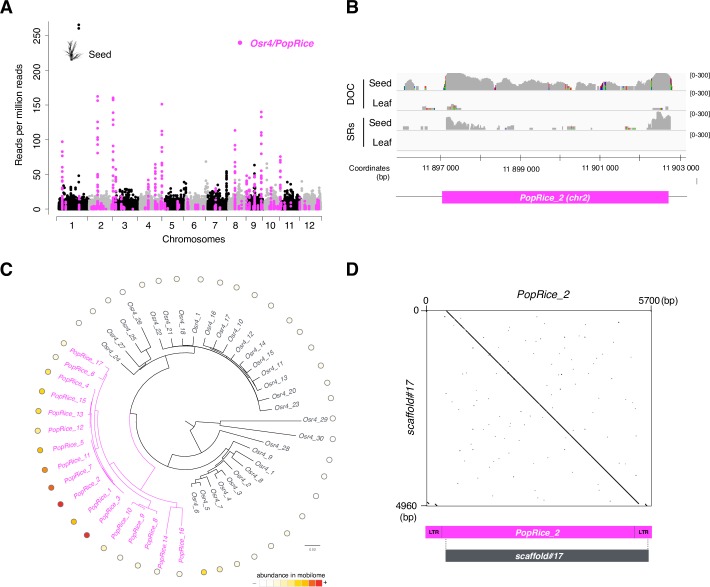
Mobilome-seq detection of a novel active retrotransposon in rice seeds. (**A**) Genome-wide analysis of mobilome-seq data identifies the *PopRice* retrotransposon family as the most represented active family in WT rice seeds. Legend as in Fig 3A. Pink dots indicate the windows corresponding to *Osr4* and *PopRice* loci. (**B**) Detail of the depth of coverage of total mapped reads and split reads abundance of the *O*. *sativa* WT seeds mobilome-seq library at the *PopRice* locus on chromosome 2 (*pink bar*) for callus and leaf mobilome-seq data. Legend as in Fig 2C. (**C**) Phylogenic tree showing that *PopRice* is a distinct subfamily of *Osr4* LTR-RT. The relative DOC calculated from two biological replicates in WT seed mobilome-seq data is indicated as a heatmap. (**D**) Dotter alignment of the scaffold #17 obtained after *de novo* assembly of WT seed mobilome-seq library and a *PopRice* element.

Epigenomic studies have revealed a release of TE transcriptional silencing during plant development [48–51]. In a first attempt to understand the possible role of TEs reactivation during plant development, we performed mobilome-seq analyses on DNA extracted from whole rice seeds. Some TE regions were significantly highly covered in this mobilome-seq library (Fig 4A and 4B, S9 Fig), most of these regions corresponding to TEs belonging to a single subfamily of *Osr4*. *Osr4* [52] is a large family of 5.7 kb-long LTR-RTs comprising 47 members in the *O*. *sativa* ssp *japonica* cv *Nipponbare* genome (Fig 4C and S3 Table). To differentiate *Osr4* active and non-active members we hereafter refer to the subfamily enriched in the seed mobilome-seq library as the *PopRice* family. The *PopRice* family is composed of 17 full-length copies in the reference genome. Some of these loci are highly covered in the seed mobilome-seq library with a DOC reaching 300X (Fig 4B), showing that some members of this subfamily are actively producing eccDNAs in wild type rice seeds. We detected SRs located on both 5’ and 3’ ends of some *PopRice* loci (Fig 4B and S9 Fig). A closer examination of reads spanning junctions has also confirmed the presence of *PopRice* eccDNAs (S10 Fig). Further sequence analyses of *PopRice* family revealed that the most active *PopRice* copies form a subgroup of 5 members (Fig 4C).

### *de novo* assembly can be used to identify the most active LTR-RTs without a reference genome

We performed *de novo* assembly of mobilome-seq libraries to determine whether *EVD*, *Tos17* and *PopRice* could be detected without mapping on a reference genome. We did not detect scaffolds corresponding to *EVD* when performing *de novo* assembly on the WT mobilome-seq library. In the Arabidopsis epi12 mobilome-seq library, *de novo* assembly resulted in three main scaffolds corresponding to *EVD* (S11 Fig). These three scaffolds all result from the assembly of a high number of reads (59,943; 49,098 and 19,424 reads per million (rpm), respectively, p-value < 0.05, negative binomial distribution). In the rice callus mobilome-seq library the most highly covered scaffold (3,906 rpm) with homology to TEs corresponded to *Tos17* (100% identity over 4,532 base pairs (bp), Fig 3D). This suggests that *de novo* assembly can be used to identify active RTs. In the seed mobilome-seq library, the most significantly covered scaffold (3,473 rpm) showed 99% of sequence identity with a *PopRice* consensus sequence over 4,960 bp (Fig 4D). Only the ends of *PopRice* were not assembled in this scaffold, likely due to the repetitive nature of LTR sequences.

### Activation of *PopRice* in the endosperm tissue

**Fig 5:**
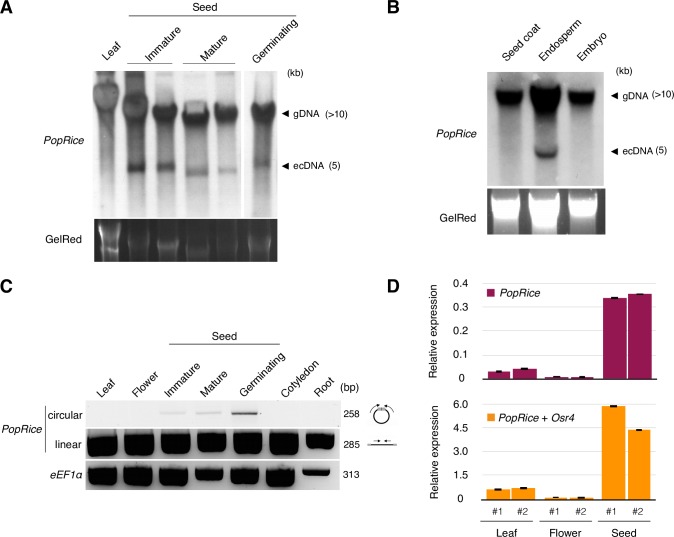
*PopRice* retrotransposons produce extrachromosomal DNA during seed development in wild type rice. (**A**) Southern blot experiment using non-digested genomic DNA extracted from WT rice leaves and seeds at different stages as indicated and detected with a *PopRice* specific probe (*gDNA*: genomic DNA, *ecDNA*: extrachromosomal DNA). The GelRed gel picture is shown as a loading control. (**B**) Southern blot experiment using non-digested genomic DNA extracted from dissected rice seed tissues as indicated and detected with a *PopRice* specific probe. Legend as in A. (**C**) Detection of *PopRice* circular forms using inverse PCR with primers localization depicted on the right (*black bar*: *PopRice* element, *arrows*: PCR primers, *grey boxes*: LTRs). Upper gel: PCR amplification of *PopRice* circles, middle gel: control PCR for *PopRice* detection, lower gel: PCR using *eEF1α* primers as control. (**D**) qRT-PCR analysis of *PopRice* and *Osr4* transcripts in WT rice leaves, flowers and mature seeds. Two pairs of primers were used: *PopRice* specific primers (top) and primers specific for the whole *Osr4* family (including *PopRice*) (bottom). The relative expression levels were calculated using *eIF-5a* as a reference, error bars indicate technical replicates, two biological replicates are shown for each tissue.

To further validate the presence of extrachromosomal DNA fragments originating from *PopRice* in WT rice seeds, we performed a Southern blot experiment using non-amplified and non-digested genomic DNA (Fig 5A). Using a *PopRice* specific probe, a signal corresponding to a 5 kb fragment was identified in genomic DNA samples extracted from seeds but not from leaves, revealing a massive accumulation of *PopRice* extrachromosomal copies in wild type seeds. A Southern blot performed on genomic DNA obtained from dissected seed tissues further revealed that *PopRice* extrachromosomal DNA could only be detected in the endosperm tissue but not in the embryo or seed coat (Fig 5B). This result was confirmed by inverse PCR assays (S12 Fig). To characterize the kinetics of *PopRice* activation during plant development we used inverse PCR to detect the presence of *PopRice* eccDNAs at different developmental stages. *PopRice* eccDNAs seemed to be specific of seed tissues from the embryo developmental stage (corresponding to immature seeds, from 3 to 5 days after pollination) to the germination, however eccDNAs were not detected in roots and cotyledons after germination (Fig 5C).

To rule out the possibility that *PopRice* circles could originate from homologous recombination between its endogenous LTR sequences, we identified mobilome-seq reads corresponding to 2LTR junctions in the seed libraries (S10 Fig). The presence of these reads confirmed that non-homologous end-joining of reverse transcription products, and not homologous recombination at the endogenous genomic location, is responsible for the formation of *PopRice* eccDNA molecules. Additionally we analyzed *PopRice* transcription, the mRNAs being the precursors of the eccDNAs. RT-qPCR assays showed that *PopRice* and *Osr4* members are highly transcribed in seeds compared to leaves and flowers (Fig 5D). The level of expression seems higher when all elements of the *Osr4* family are considered suggesting that the whole *Osr4* family is transcriptionally active, although only *PopRice* eccDNAs could be detected (Fig 4C).

## Discussion

In all eukaryotic organisms, eccDNA molecules are ubiquitous elements and constitute an heterogenous population of circular molecules that can originate from repeats such as rDNA clusters through homologous recombination [34,53] or from active TEs (through circularization of linear extrachromosomal forms). We took advantage of the detection of eccDNAs by next generation sequencing (NGS) to explore the extrachromosomal circular mobilome in plants. As a proof of concept we analyzed samples from *A*. *thaliana* and *O*. *sativa* material for which actively proliferating TEs had previously been characterized [44,45]. Identification of two well-characterized active LTR-RTs, *EVD* and *Tos17* in an *A*. *thaliana* hypomethylated line and in rice callus tissue, respectively, confirmed that this method is efficient to capture actively proliferating retrotransposons in plants. The detection of rDNA circles validates the enrichment of eccDNAs in our libraries and thus constitutes another positive control. Moreover our observations suggest that some TEs might form different circles where SRs spread into internal regions of TEs reflecting a possible heterogeneity of these extrachromosomal circles. The mobilome-seq strategy exploits the advantages of NGS and requires a low sequencing coverage for each library. Indeed only a minor fraction of the genome is sequenced, opening the future possibility of applying the technique to very large genomes, for which resequencing techniques are not affordable and technically challenging. Furthermore, the *de novo* assembly analyses might represent a precious and powerful method to study the active mobilome of species for which a reference genome is lacking.

Developmental relaxation of TE control has been documented in plant tissues accompanying the gametes: vegetative nucleus for the pollen and endosperm for the ovary [51]. In rice, DNA methylome analyses revealed a global hypomethylation in the endosperm [50,54,55] confirming previous results in Arabidopsis [49,56,57]. This suggests that TE activity could be increased in these tissues; however, to our knowledge, only the proliferation of a DNA-TE Mule element has been documented so far in the *A*. *thaliana* pollen [48]. Here, our seed mobilome-seq analyses reproducibly revealed that the *PopRice* family of autonomous LTR-RTs produces extrachromosomal copies. These copies can be detected on a Southern blot analysis of untreated genomic DNA showing that *PopRice* extrachromosomal copies accumulate in wild type rice endosperm. Further studies will help evaluating the proliferation of *PopRice* in the endosperm genome. *PopRice* transcripts could be detected in seeds suggesting that eccDNAs indeed originate from reverse transcription of these transcripts. Genomic imprinting could explain the transcriptional activity of some TEs in the endosperm. According to a study by Luo *et al*. [58], only two relatively ancient copies (*PopRice_16* and *Osr4_28*) are localized in introns of paternally imprinted genes (LOC_Os11g09329 and LOC_Os08g24420, respectively) suggesting that imprinting might not be the only trigger for *PopRice/Osr4* transcriptional activation in the endosperm. Recently, Cheng *et al*. have shown that members of the *Osr4* LTR-RT family could retrotranspose in *oscmt3* mutants, affected in a chromomethylase involved in DNA methylation, through genome resequencing [59]. Interestingly all neo-insertions are due to the *PopRice* subfamily suggesting that this subfamily contains all potentially active members and that they are under the epigenetic control of OsCMT3. This transgenerational control is reminiscent of the regulation of *Onsen*, an *A*. *thaliana* LTR-RT that produces eccDNA molecules after heat stress. However in the case of *Onsen* transgenerational neo-insertions are only detected in mutants affected in the RNA-directed DNA methylation (RdDM) pathway, but not in the CMT3 pathway [60]. The precise role of the RdDM pathway in the transgenerational control of these LTR-RTs neo-insertions is not yet elucidated [61].

Using a newly developed method to sequence and identify eccDNAs originating from TEs, we have characterized a yet unexplored fraction of plant DNA. This study revealed the reactivation of an endosperm-specific LTR-RT in rice. This LTR-RT family seems to be under the control of both epigenetic and post-transcriptional regulation. Furthermore the identification of this only LTR-RT family active in the endosperm suggests that the global hypomethylation occurring in this tissue is not sufficient to trigger a massive reactivation of TEs. By giving an insight into actively proliferating retrotransposons in plants, the mobilome-seq approach is likely to expand our understanding of TE activity in plants and of their putative contribution in response to stress and during plant development.

## Materials and methods

### Plant material

*Arabidopsis thaliana* WT ecotype Columbia-0 and epiRIL12 plants from the eighth generation [43] were grown in soil under a 16h/8h (light/dark) cycle after 2 days at 4°C for stratification. Florets and 1–2 cm green siliques were harvested 3 days to 2 weeks after pollination, respectively. *Oryza sativa* ssp. *japonica* cv. *Nipponbare* rice plants were cultivated in a growth chamber (Percival, USA) under a 12h light-dark cycle (12h-28°C/12h-26°C) and with a relative humidity of 80% during the day and 70% during the night. The light intensity varied gradually in 40 min at the beginning and end of the day. Grain material was harvested 3 to 5 to 15 days after pollination for the immature and mature stage, respectively. Seeds were germinated in the dark on a humid Whatman paper for 5 days before harvest. Dissection of seeds was performed under the binocular on mature seeds. Callus material was previously described [21].

### DNA extraction

For each plant sample, total DNA was extracted using the plant DNeasy mini kit (Qiagen) according to the manufacturer’s instructions. A DNA pre-extraction was performed for rice grains to optimize DNA quantity and quality. Grains were grinded in an extraction buffer (Tris-HCl pH8, NaCl 250mM, EDTA 50mM, 0.2% SDS) and were incubated 30 min at 65°C. DNA samples were precipitated with 0.7 volume of isopropanol and the DNA pellet was directly resuspended in the plant DNeasy mini buffer (Qiagen).

### Extrachromosomal circular DNA enrichment

To remove large genomic linear fragments 5μg of genomic DNA for each sample were purified using a Geneclean kit (MPBio) according to the manufacturer’s instructions. eccDNA was isolated from 28μl of the GeneClean product using the PlasmidSafe DNase (Epicentre) according to the manufacturer’s instructions, except that the 37°C incubation was performed for 17h. The PlasmidSafe exonuclease digests double-stranded linear DNA to deoxynucleotides while leaving circular DNA intact. DNA samples were precipitated by adding 0.1 volume of 3M sodium acetate (pH 5.2), 2.5 volumes of ethanol and 1 μl of glycogen (Fisher) and incubating overnight at -20°C. The precipitated circular DNA was amplified by random RCA using the Illustra TempliPhi kit (GE Healthcare). For this, the DNA pellet was directly resuspended in the Illustra TempliPhi Sample Buffer, and the reaction was performed according to the manufacturer’s instructions except that the incubation was performed for 65h at 28°C. One tenth of each amplified DNA sample was digested with restriction enzymes and loaded on an agarose gel electrophoresis to control the DNA quality and amplification. Then, the DNA concentration was determined using the DNA PicoGreen kit (Invitrogen) following the manufacturer’s instructions, the fluorescence being read using a LightCycler480 (Roche). The samples were diluted to a final concentration of 0.2 ng/μl in order to prepare the libraries for sequencing.

### Libraries preparation and sequencing

One nanogram of DNA from each sample was used to prepare the libraries using the Nextera XT library kit (Illumina) according to the manufacturer’s user guide. Each mobilome-seq library was amplified by 12 cycles of PCR using index primers. DNA quality and concentration were determined using a high sensitivity DNA Bioanalyzer chip (Agilent Technologies). Samples were pooled and loaded onto a flow cell and 2x250 nucleotides paired-end sequencing was performed using the MiSeq platform (Illumina). Up to twelve mobilome-seq libraries were pooled into one run and an average of 1 million reads per library were obtained (S1 and S2 Tables). Illumina reads were collected for the analysis as FASTQ files.

### Data analysis

To analyze the sequencing reads we anticipated that the eccDNAs of interest originating from mobile TEs should represent a very small fraction of the genome and consequently that the loci from where these eccDNAs were produced should be highly covered. Furthermore, as these molecules are circular, reads spanning the junction of the circles should not map properly on the reference genome because such junctions do not exist in the chromosomes. However, these reads might map on two different locations (start and end of the element). Thus the eccDNAs of interest should fit to the two following criteria: (1) high DOC and (2) presence of SRs when mapped to a reference genome. Finally, due to the repetitive nature of TEs, we reasoned that the read-mapping coverage could be less sensitive for large TE families as reads could be dispersed amongst related TE copies. Therefore we should be able to identify the most abundant eccDNAs by analyzing highly covered scaffolds after *de novo* assembly.

### Read mapping

Quality control of FASTQ files was evaluated using the FastQC tool (version 0.10.1 www.bioinformatics.babraham.ac.uk/projects/fastqc). To remove any read originating from organelle circular genomes, reads were mapped against the mitochondria (NCBI GenBank Y08501.2 for *A*. *thaliana*; GenBank NC_011033 for O. *sativa*) and chloroplast genomes (GenBank AP000423.1 for *A*. *thaliana*; GenBank X15901 for *O*. *sativa*) using the program BOWTIE2 version 2.2.2 [62] with—sensitive local mapping. Unmapped reads were considered for the next analysis and were mapped against a genome of reference, TAIR10 (The Arabidopsis Information Resource, http://www.arabidopsis.org) for *A*. *thaliana*, IRGSP1.0 (International Rice Genome Sequencing Project version 5 http://rgp.dna.affrc.go.jp/E/IRGSP/Build5.html) for *O*. *sativa*. The parameters used for the mapping were as follows:—sensitive local mapping, no multiple-mappings (-k 1) so that only the best hit is kept per read-pair. DNA from both mitochondria and chloroplast genomes are integrated in nuclear genomes. To completely eliminate these regions from our data, sequencing reads were simulated from organelle genome using the dwgsim program (version 0.1.10) and the .*fasta* files were mapped against the corresponding reference genome. A total of 816,300 bp and 1,697,400 bp were masked in *A*. *thaliana* and *O*. *sativa*, respectively, and TE containing regions cover 24,786,000 bp and 194,224,800 bp in *A*. *thaliana* and *O*. *sativa*, respectively. A .*bam* file with all genome regions corresponding to organelle integrated sequences was obtained for each species and was used to filter our alignment files using the intersect module of BEDTools version 2.21.0 (option -v). Finally, for each library, a .*bam* alignment file corresponding to enriched genomic regions was considered for statistical analysis and visualized with the Integrative Genomics Viewer (IGV) software (https://www.broadinstitute.org/igv/home) and Circos [63].

### Statistical analysis

For each species, the reference genome was split into consecutive windows of 100bp for each library and the coverageBED module of BEDTools was used to determine the read coverage depth of these non-overlapping windows. The coverage data was normalized by the total number of reads which mapped on the genome expressed in rpm and statistical analysis was performed on these files. First we determined covered regions using the Poisson distribution that best fits our data with a p-value <10^−5^ for each library. All uncovered regions were removed from our coverage files. On the covered regions we applied a negative binomial distribution to identify peaks of higher coverage with a p-value <10^−3^. Finally, regions corresponding to the peaks were selected and annotated using .*gff* files (S5 Table). All statistical analysis and graphics were performed using R (Rstudio package version 0.98.1091, www.r-project.org/).

### *de novo* assembly

Mobilome-seq reads were assembled *de novo* using the A5-miseq pipeline [64]. For each library, .*fasta* and .*bam* files were obtained and the idxstats module of SAMtools was used to determine the read number corresponding to each assembled scaffold. The coverage data was normalized by the total number of reads used for the *de novo* assembly expressed in million reads (rpm). We applied a negative binomial distribution to identify significantly covered scaffolds (p-value < 0.05). Filtered scaffolds were annotated using a BLAST analysis (-p -m 8) against organelle genomes and a TE database allowing for one hit per scaffold (-b 1 -v 1 options) and for an e-value < 10^−2^. For *A*. *thaliana* we used the TE database based on TAIR10 annotation and established by H. Quesneville (www.arabidopsis.org); for *O*. *sativa* we used an in house curated database (www.panaudlab.org). Resulting hits were filtered to keep only scaffolds with a HSP ≥ 100bp.

### Split-reads detection

Reads spanning 2 LTR junctions constitute an evidence of a circular TE and were detected using a SR mapping strategy. Reads were aligned against the reference genomes using the segemehl software [65] with the following parameters: -S (SR mapping) -A 95 (accuracy of 95%) -U 24 (minimum score of 24) -Z 25 (minimum length of 25) -W 95 (alignment covered on 95% of the read). Split reads were collected from .*bam* files based on the FLAG field of each read, using the view module of SAMtools. Therefore, only reads which were not mapped in a proper pair (-f 14) and which have multiple primary alignments (-F 256) were considered as SR. The coverageBED module of BEDTools was used to determine the read coverage depth of these SR .*bam* files were visualized with IGV.

### Candidate TEs

To determine TE loci of interest in each library, we first filtered the .*bam* files obtained from bowtie2 mapping (for the DOC) for TEs covered for 90% of their length and with a DOC >10 rpm. Using the .*bam* files obtained from segemehl mapping we selected the TE loci with a SR coverage >10 rpm. We selected the TE loci fitting both criteria and which length is > 100bp (S6 Table). The TE loci were visualized with Circos [63]. The in-house developed code for DOC and SR detection and for the establishment of the candidate TE list can be accessed upon request.

### Southern blot analysis

Total genomic DNA was extracted using the CTAB method [66] and samples were loaded on a 0.8% agarose gel and transferred onto Hybond-N+ nylon membrane (GE Healthcare). The Southern blot on *A*. *thaliana* material was performed as previously described [44] using a radioactive probe. The Southern blot on *O*. *sativa* material was performed using a non-radioactive probe and the hybridization signal was detected with the DIG system (Roche) following the manufacturer’s instructions. Stringency washes were performed at 65°C in 0.5X SSC. Probes were amplified from genomic DNA by PCR using primers listed in the S4 Table. The Southern blots on Fig 5A and 5B were repeated twice using biological replicates.

### Transcription analysis

Total RNAs were isolated from leaves, flowers and seeds using the Tri-reagent (MRC) according to the manufacturer's instructions. RNAs were treated with DNAse from RQ1 kit (Promega) and 1.25 μg were reverse-transcribed into cDNAs using the GoScript kit (Promega). Analyses by quantitative real-time PCR (qRT-PCR) were established using 7 to 35 ng of cDNA. qRT-PCRs were run on a LightCycler 480 (Roche) using Takyon No Rox SYBR MasterMix dTTP Blue Kit (Eurogentec) according to the manufacturer’s instructions. The qRT-PCR conditions were the following: a first denaturation step at 95°C for 5 min followed by 40 cycles at 95°C for 15s, an annealing and elongation step at 60°C for 60s, and a melting curve analysis at 95°C for 10s, 60°C for 10s, an increase of 0.04°C per second until 95°C and a final step of cooling at 40°C for 30s. Two biological replicates were analyzed for each tissue. *PopRice* and *Osr4* expression levels relative to *eIF-5a* [67] were calculated using the formula: 2^-(mean (CT PopRice—CT internal references))^. Primers were used with a concentration of 2μM and primers details are given in the S4 Table and S13 Fig.

### PCR validations

PCR reactions were performed using 2 μl of DNA (before or after the RCA amplification) in a final volume of 15 μl, using the GoTaq polymerase (Promega). All primer pairs were designed using Primer3 (www.primer3.ut.ee) and quality-checked using OligoCalc (www.basic.northwestern.edu/biotools/oligocalc.html) and BLAST (www.ncbi.nlm.nih.gov/BLAST/). Target sequences and primers used are shown in S4 Table. The PCR conditions were the following: a first denaturation step at 95°C for 5 min followed by 30 cycles at 95°C for 30s, an annealing step (temperature details in S4 Table) for 30s, an elongation step at 72°C for 20 seconds, and a final extension step at 72°C for 5 min. 8 μl of PCR products were deposited on a 1,5% agarose gel and run at 135mV for 30 min. DNA was stained using a GelRed dye (Biotium). Gel pictures were obtained using an UGenius gel imaging system (Syngene). All PCR assays were repeated at least twice using biological replicates.

### Identification of *PopRice* subfamily

To determine the evolutionary story of *PopRice* elements, a consensus sequence of *PopRice* was used to detect by BLAST all LTR-RTs from the IRGSP-1.0 reference genome belonging to the same family (HSP>4000bp, minimum of 70% of identity, e-value < e^-50^). All selected sequences were aligned with MAFFT (http://mafft.cbrc.jp/alignment/server/). Alignments were analyzed using SEAVIEW (http://doua.prabi.fr/software/seaview) and all incomplete elements were removed. 47 elements were selected for the *Osr4* family (comprising *PopRice* sequences) and a phylogenetic tree was built with PhyML and visualized with FigTree (http://tree.bio.ed.ac.uk/software/figtree/).

### Accession number

Sequencing data generated in this study have been deposited at the European Nucleotide Archive (ENA, www.ebi.ac.uk/ena) under the accession number PRJEB13537.

## Supporting information

S1 Fig Lifecycle of LTR-RTs. The retrotransposition cycle is composed of different steps. The 5’LTR contains a RNA polymerase II promoter sequence and marks the start of transcription (**1**) and in contrast the 3’ LTR indicates the stop and the polyadenylation signal. LTR transcripts are used both as a matrix for translation (**2**, **3**) and for reverse transcription (**6–8**). In the cytoplasm, the polyprotein is self-cleaved into 4 proteins (**3**): a reverse transcriptase (RT; *green dot*), a RNaseH (*yellow dot*), an aspartic proteinase (AP; *purple dot*) and an integrase (IN; *blue dot*). The interaction between some gag proteins induces the protection of the transcript and of the 4 proteins in a virus-like particle (VLP) (**4**, **5**). The binding of a host tRNA on the primer binding site (PBS) flanking the 3’ end of the 5’ LTR initiate the reverse transcription of the transcript into DNA via the RT (**6**). The RNAseH degrades the RNA template (**7**) and the complementary strand is reverse transcribed (**8**). The newly synthesized ecDNA copy associated with IN (**9**) migrates into the nucleus using unknown mechanisms (**10**). This ecDNA can lead to a new insertion in the host genome (**11**) or alternately can be recognized by DNA repair mechanisms (either non-homologous end-joining (NHEJ) or homologous recombination) to form eccDNA molecules (**12**).
(PDF)

S2 Fig Detection of eccDNAs originating from ribosomal DNA repeats in *A*. *thaliana*. (**A**) Abundance of reads mapping at TE-annotated and rDNA loci in the *A*. *thaliana* WT mobilome-seq library. Each dot represents the normalized coverage per million mapped reads per all TE-containing (*black circles*) or rDNA containing (*red dots*) 100bp windows obtained after aligning the sequenced reads on the reference genome. (**B**) Abundance of reads mapping at TE-annotated and rDNA loci in the *A*. *thaliana* epi12 mobilome-seq library. Legend as in (A).
(PDF)

S3 Fig Analysis of *A*. *thaliana* mobilome-seq libraries. (**A**) Abundance of reads mapping at TE-annotated loci in the *A*. *thaliana* WT mobilome-seq library. (**B**) Statistical analysis of the WT mobilome-seq library presented in (A). (**C**) Statistical analysis of the epi12 mobilome-seq library presented in Fig 2B. Legend as in Fig 2B.
(PDF)

S4 Fig Split read (SR) analysis of *A*. *thaliana* mobilome-seq libraries. (**A**) Abundance of SRs mapping at TE-annotated loci in the *A*. *thaliana* WT mobilome-seq library. (**B**) Abundance of SRs mapping at TE-annotated loci in the *A*. *thaliana* epi12 mobilome-seq library. Legend as in Fig 2B.
(PDF)

S5 Fig *EVD* forms eccDNAs in *A*. *thaliana* epi12 line. Example of a SR identified in the *A*. *thaliana* epi12 mobilome-seq library spanning the junction of the 2LTR-circle corresponding to *EVD* aligned with an artificial junction corresponding to the 3’ part of the 3’LTR (*red box*) fused to the 5’ part of the 5’LTR (*yellow box*).
(PDF)

S6 Fig Comparison between mobilome and transcriptome data. A circos plot showing, from outermost to innermost track, scatter plots for (i) split reads coverage per million reads per TE locus (SRs, *red track*), (ii) total coverage per million reads per TE locus (DOC, *blue track)* and (iii) transcriptome coverage at TEs (TR, *green track*). Transcriptome data are presented as the log_2_ of fold change in epi12 versus WT at significantly upregulated TE loci [47]. The tracks are scaled separately. The chromosome sizes are indicated in megabase pairs. For mobilome-seq data the names of TE loci that are covered on 90% of their length with both a DOC and SR value >5 reads per million reads are indicated. Data used for this plot are available in S7 Table.
(PDF)

S7 Fig Analysis of the *O*. *sativa* WT callus mobilome-seq libraries. (**A**) Statistical analysis of the mobilome-seq library presented in Fig 3A. Legend as in Fig 3A. (**B**) Abundance of SR mapping at TE-annotated loci in the WT callus mobilome-seq library.
(PDF)

S8 Fig *Tos17* and *Lullaby* form eccDNAs in rice calli. (**A**) Example of split read spanning the perfect junction of the 2LTR-circle corresponding to *Tos17*. Legend as in S5 Fig. (**B**) Example of split read spanning the perfect junction of the 2LTR-circle corresponding to *Lullaby*.
(PDF)

S9 Fig Analysis of the *O*. *sativa* seed mobilome-seq libraries. (**A**) Statistical analysis of the mobilome-seq library presented in Fig 4A. Legend as in Fig 4A. (**B**) Abundance of split reads mapping at TE-annotated loci in the *O*. *sativa* WT seed mobilome-seq library.
(PDF)

S10 Fig *PopRice* retrotransposon forms eccDNAs in rice seeds. (**A**) Example of a split read spanning the perfect junction of the 2LTR-circle corresponding to *PopRice*. (**B**) Example of a split read spanning the imperfect junction of the 2LTR-circle corresponding to *PopRice*. A primer binding site (PBS) sequence is highlighted in blue. The PBS is normally found after the 5’LTR in a linear *PopRice*. Legend as in S5 Fig.
(PDF)

S11 Fig *De novo* assembly of *EVD* eccDNAs. Example of scaffolds obtained after *de novo* assembly of epi12 mobilome-seq library and corresponding to *EVD*. The presence of many scaffolds (and not only one) suggests that *EVD* forms a complex population of circles.
(PDF)

S12 Fig Detection of *PopRice* eccDNAs by PCR. Circular forms of *PopRice* are specifically detected in the dissected rice endosperm using inverse PCR. Legend as in Fig 5C. PCR using *eEF1α* primers is used as a loading control.
(PDF)

S13 Fig Schemes depicting the localization of primers and probes used in this study for the analyzed retrotransposons. (PDF)

S1 Table Characteristics of the *A*. *thaliana* mobilome-seq libraries. (PDF)

S2 Table Characteristics of the *O*. *sativa* mobilome-seq libraries. (PDF)

S3 Table Localization of *PopRice* and *Osr4* elements in the *O*. *sativa* ssp. *japonica* cv. Nipponbare reference genome.
(PDF)

S4 Table List of primers used in this study. (PDF)

S5 Table Significant coverage values. For each library, the number of mapped reads per million per 100bp window is indicated with P-value < 10^−3^ (CHR: chromosome, BP: base pair start coordinate of the 100bp window).
(PDF)

S6 Table Full mobilome-seq data. For each library, the peaks corresponding to candidate TEs are listed.
(PDF)

S7 Table Data used for the S6 Fig circos plot. (XLS)

S8 Table Data used for the plot in Fig 2. (XLS)
