# Supplementary figures and images for: Sequencing the extrachromosomal circular mobilome reveals retrotransposon activity in plants
Source: PLoS Genet. 2017 Feb 17;13(2):e1006630. doi: 10.1371/journal.pgen.1006630 (PMC5338827; doi:10.1371/journal.pgen.1006630)

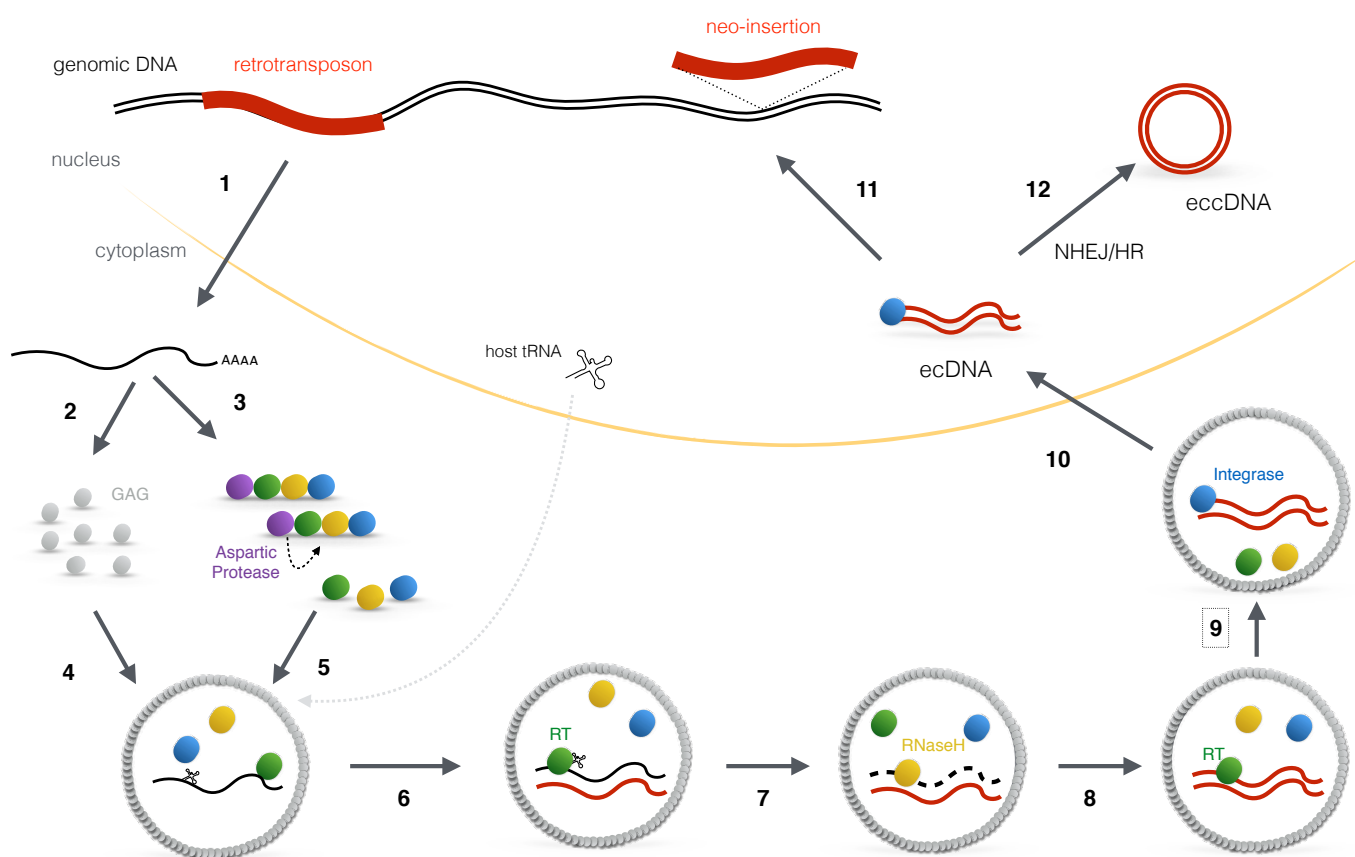

**Supplementary Figure 1**

Supplement: S1 Fig — The retrotransposition cycle is composed of different steps. The 5’LTR contains a RNA polymerase II promoter sequence and marks the start of transcription (1) and in contrast the 3’ LTR indicates the stop and the polyadenylation signal. LTR transcripts are used both as a matrix for translation (2, 3) and for reverse transcription (6–8). In the cytoplasm, the polyprotein is self-cleaved into 4 proteins (3): a reverse transcriptase (RT; green dot), a RNaseH (yellow dot), an aspartic proteinase (AP; purple dot) and an integrase (IN; blue dot). The interaction between some gag proteins induces the protection of the transcript and of the 4 proteins in a virus-like particle (VLP) (4, 5). The binding of a host tRNA on the primer binding site (PBS) flanking the 3’ end of the 5’ LTR initiate the reverse transcription of the transcript into DNA via the RT (6). The RNAseH degrades the RNA template (7) and the complementary strand is reverse transcribed (8). The newly synthesized ecDNA copy associated with IN (9) migrates into the nucleus using unknown mechanisms (10). This ecDNA can lead to a new insertion in the host genome (11) or alternately can be recognized by DNA repair mechanisms (either non-homologous end-joining (NHEJ) or homologous recombination) to form eccDNA molecules (12). (PDF) [file pgen.1006630.s001.pdf]

**A**

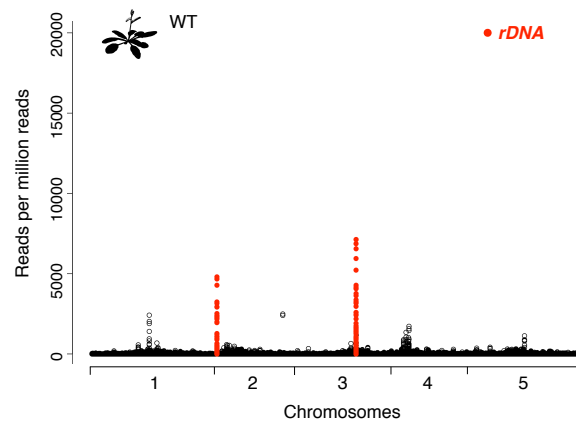

**B**

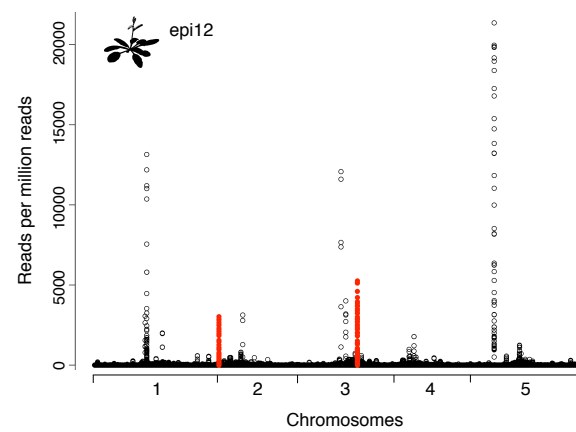

Supplementary Figure 2

Supplement: S2 Fig — (A) Abundance of reads mapping at TE-annotated and rDNA loci in the A. thaliana WT mobilome-seq library. Each dot represents the normalized coverage per million mapped reads per all TE-containing (black circles) or rDNA containing (red dots) 100bp windows obtained after aligning the sequenced reads on the reference genome. (B) Abundance of reads mapping at TE-annotated and rDNA loci in the A. thaliana epi12 mobilome-seq library. Legend as in (A). (PDF) [file pgen.1006630.s002.pdf]

**A**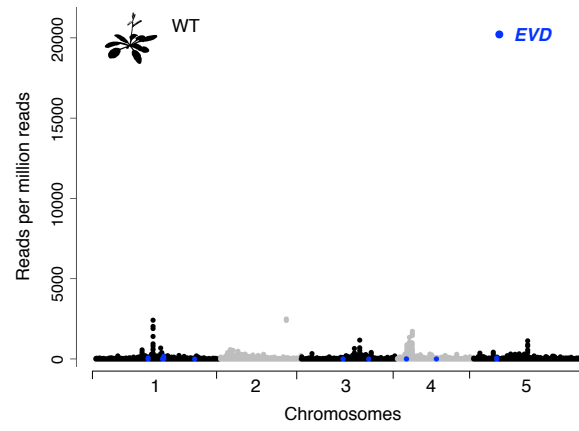**B**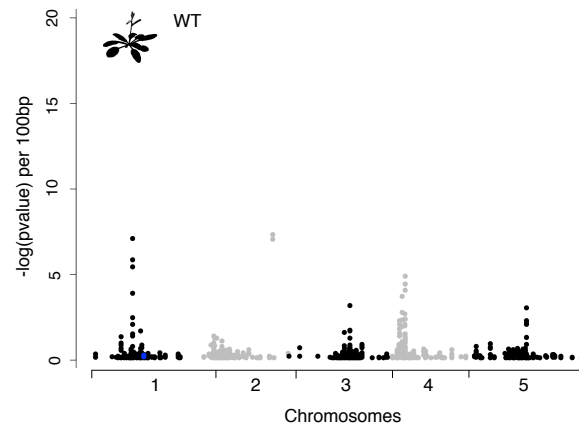**C**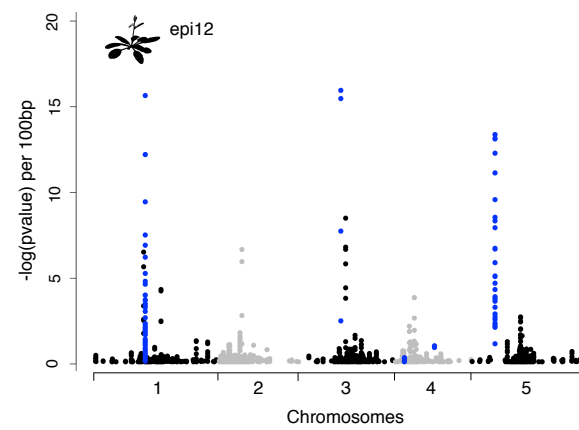

Supplementary Figure 3

Supplement: S3 Fig — (A) Abundance of reads mapping at TE-annotated loci in the A. thaliana WT mobilome-seq library. (B) Statistical analysis of the WT mobilome-seq library presented in (A). (C) Statistical analysis of the epi12 mobilome-seq library presented in Fig 2B. Legend as in Fig 2B. (PDF) [file pgen.1006630.s003.pdf]

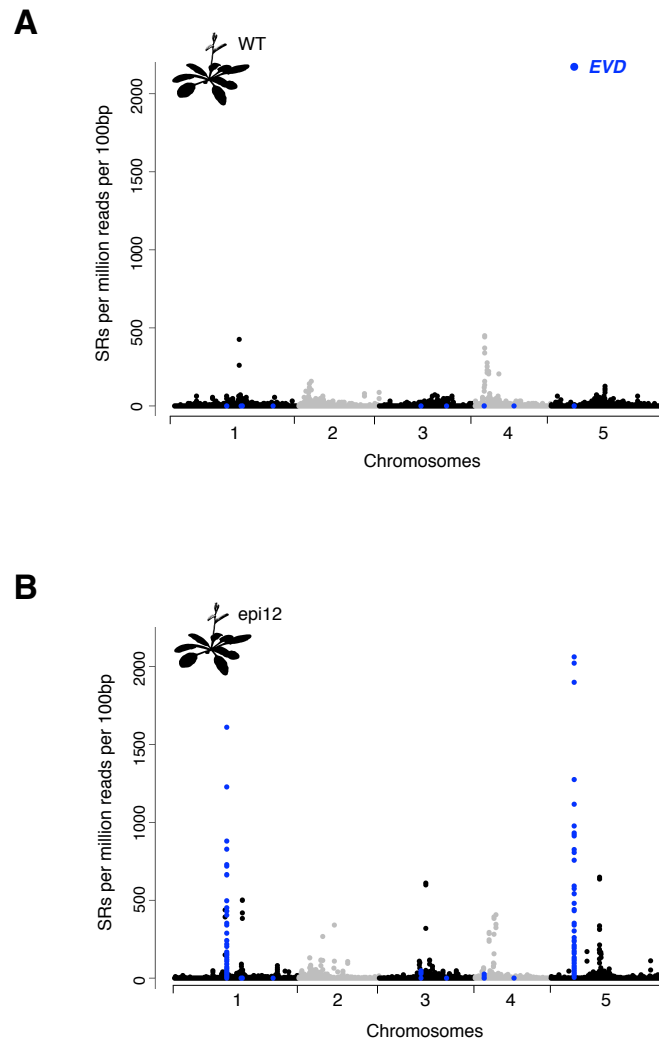

Supplementary Figure 4

Supplement: S4 Fig — (A) Abundance of SRs mapping at TE-annotated loci in the A. thaliana WT mobilome-seq library. (B) Abundance of SRs mapping at TE-annotated loci in the A. thaliana epi12 mobilome-seq library. Legend as in Fig 2B. (PDF) [file pgen.1006630.s004.pdf]

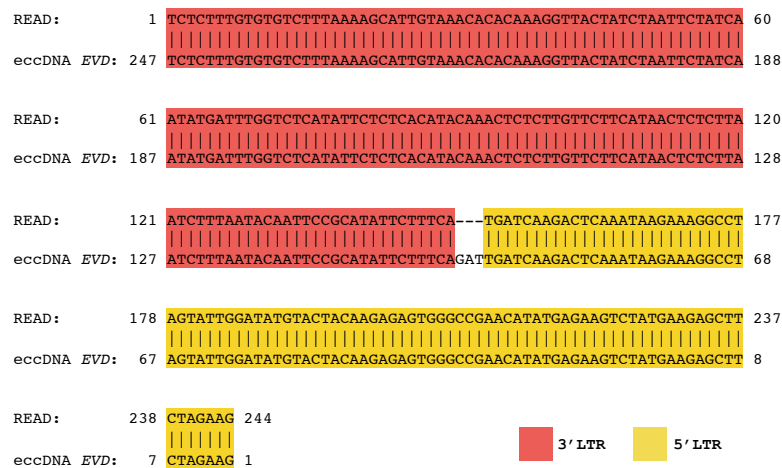

Supplementary Figure 5

Supplement: S5 Fig — Example of a SR identified in the A. thaliana epi12 mobilome-seq library spanning the junction of the 2LTR-circle corresponding to EVD aligned with an artificial junction corresponding to the 3’ part of the 3’LTR (red box) fused to the 5’ part of the 5’LTR (yellow box). (PDF) [file pgen.1006630.s005.pdf]

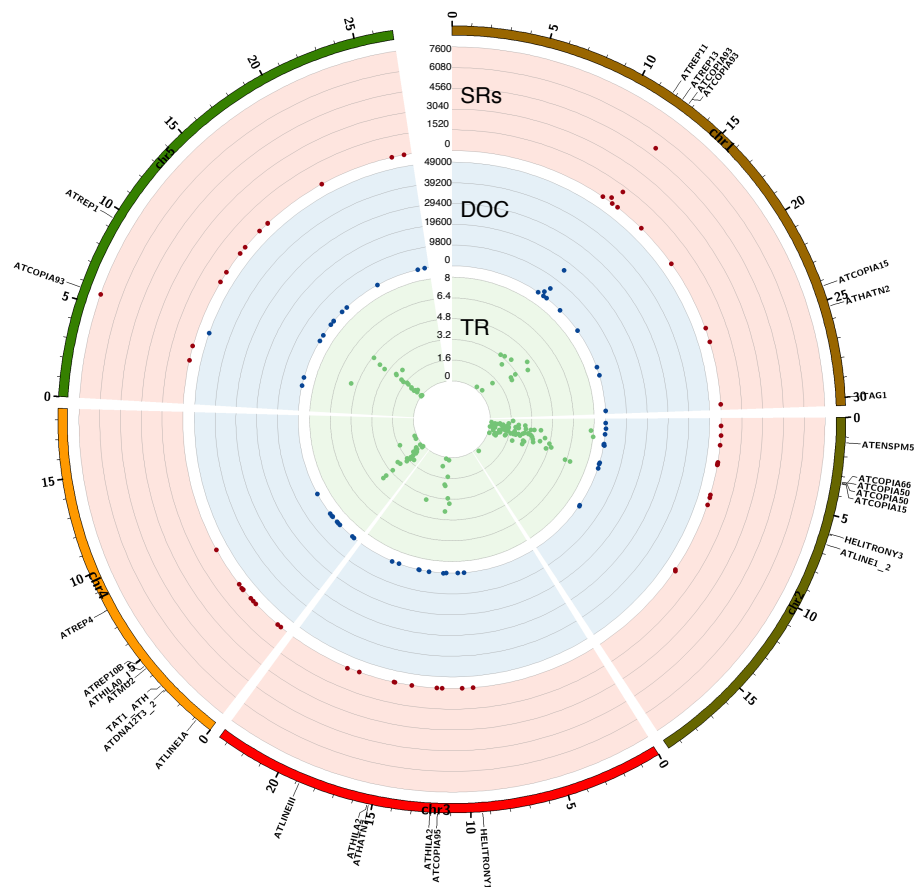

Supplementary Figure 6

Supplement: S6 Fig — A circos plot showing, from outermost to innermost track, scatter plots for (i) split reads coverage per million reads per TE locus (SRs, red track), (ii) total coverage per million reads per TE locus (DOC, blue track) and (iii) transcriptome coverage at TEs (TR, green track). Transcriptome data are presented as the log2 of fold change in epi12 versus WT at significantly upregulated TE loci [47]. The tracks are scaled separately. The chromosome sizes are indicated in megabase pairs. For mobilome-seq data the names of TE loci that are covered on 90% of their length with both a DOC and SR value >5 reads per million reads are indicated. Data used for this plot are available in S7 Table. (PDF) [file pgen.1006630.s006.pdf]

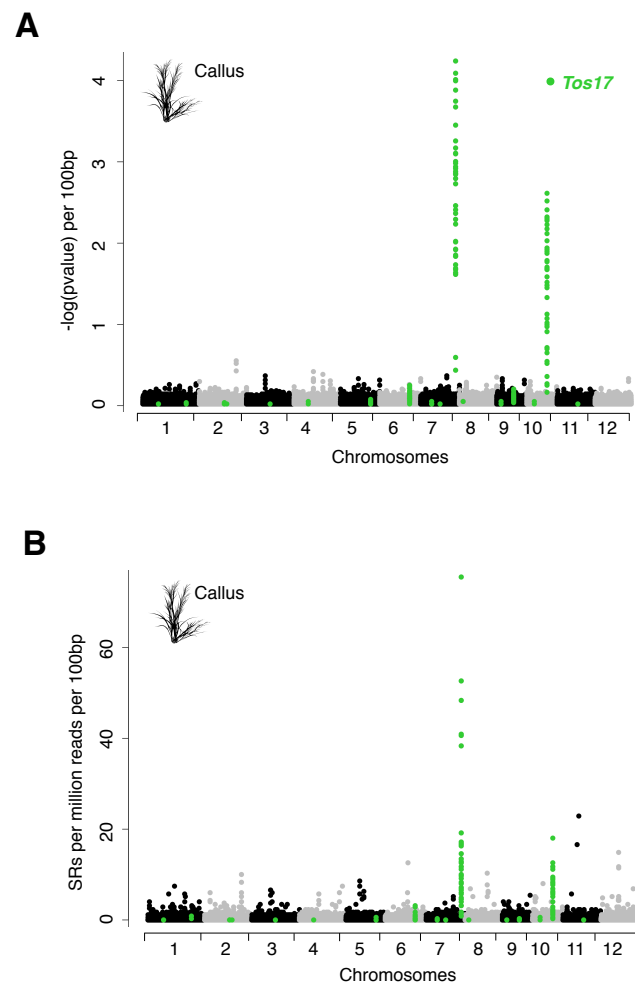

Supplementary Figure 7

Supplement: S7 Fig — (A) Statistical analysis of the mobilome-seq library presented in Fig 3A. Legend as in Fig 3A. (B) Abundance of SR mapping at TE-annotated loci in the WT callus mobilome-seq library. (PDF) [file pgen.1006630.s007.pdf]

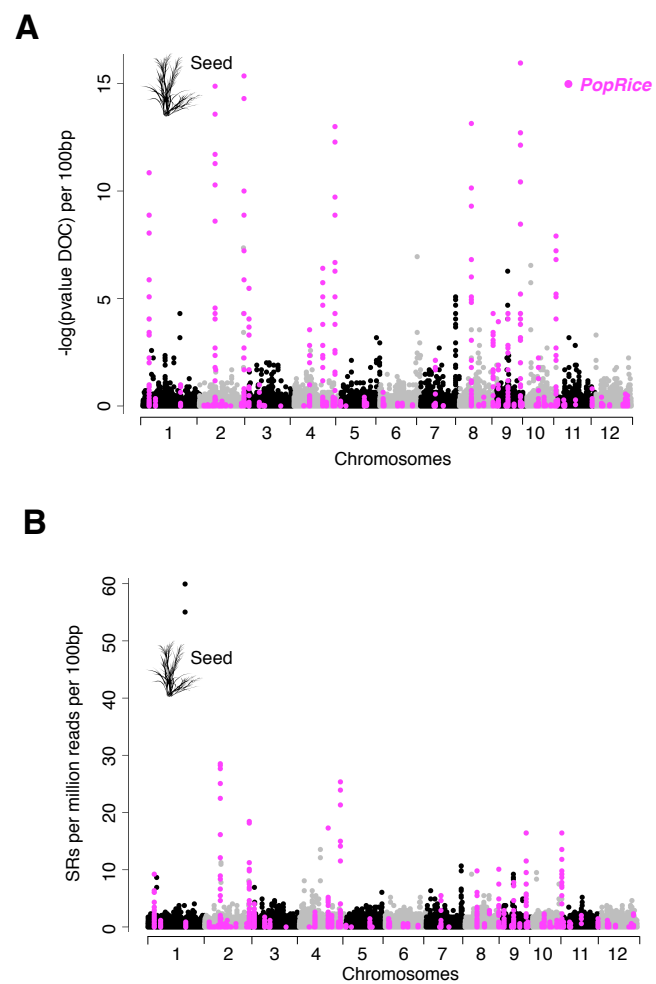

Supplementary Figure 9

Supplement: S9 Fig — (A) Statistical analysis of the mobilome-seq library presented in Fig 4A. Legend as in Fig 4A. (B) Abundance of split reads mapping at TE-annotated loci in the O. sativa WT seed mobilome-seq library. (PDF) [file pgen.1006630.s009.pdf]

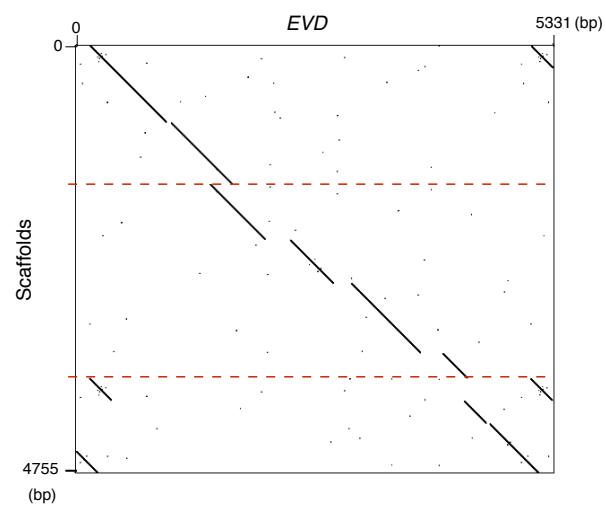

**Supplementary Figure 11**

Supplement: S11 Fig — Example of scaffolds obtained after de novo assembly of epi12 mobilome-seq library and corresponding to EVD. The presence of many scaffolds (and not only one) suggests that EVD forms a complex population of circles. (PDF) [file pgen.1006630.s011.pdf]

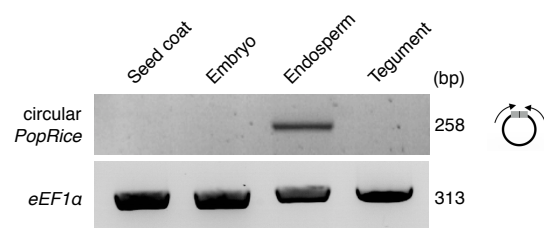

**Supplementary Figure 12**

Supplement: S12 Fig — Circular forms of PopRice are specifically detected in the dissected rice endosperm using inverse PCR. Legend as in Fig 5C. PCR using eEF1α primers is used as a loading control. (PDF) [file pgen.1006630.s012.pdf]

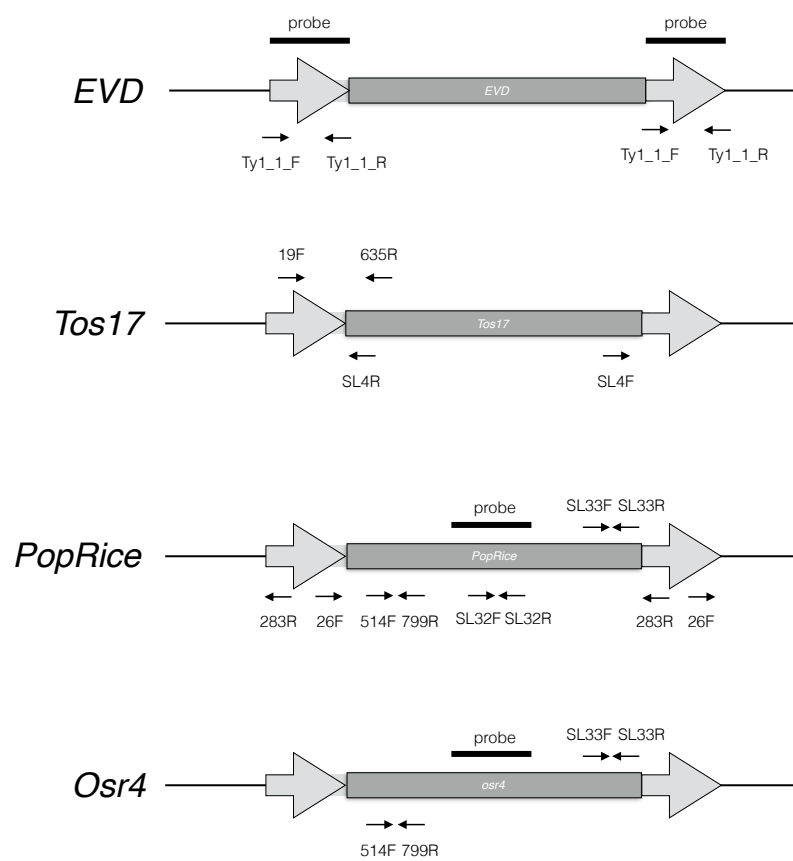

**Supplementary Figure 13**

Supplement: S13 Fig — (PDF) [file pgen.1006630.s013.pdf]
