# Supplementary material for: Sequencing the extrachromosomal circular mobilome reveals retrotransposon activity in plants
Source: PLoS Genet. 2017 Feb 17;13(2):e1006630. doi: 10.1371/journal.pgen.1006630 (PMC5338827; doi:10.1371/journal.pgen.1006630)
Supplement: S8 Fig — (A) Example of split read spanning the perfect junction of the 2LTR-circle corresponding to Tos17. Legend as in S5 Fig. (B) Example of split read spanning the perfect junction of the 2LTR-circle corresponding to Lullaby. (PDF) [file pgen.1006630.s008.pdf]

A

```
Read:           40 TCCACCTTGAGTTTGAAGGGGGGTGTTAAATATATATACAAGCTAATGTACTGTATAGTT 99
                   |||||||||||| |||||||||||||||||||||||||||||||||||||||| ||||||
eccDNA Tos17:  251 TCCACCTTGAGTGTGAAGGGGGGTGTTAAATATATATACAAGCTAATGTACTGGATAGTT 192

Read:          100 GGCCCATGTCCAGCCCATCGGATGTCCAGCCCATTGGATCTTGTATCTTGTATATACTTC 159
                   ||||||||||||||||||||||||||||| ||||||||||||||||||||||||||||||
eccDNA Tos17:  191 GGCCCATGTCCAGCCCATCGGATGTCCAGTCCATTGGATCTTGTATCTTGTATATACTTC 132

Read:          160 TCTATTGCTAATACTATTGTTAGGTTGCAAGTTAGTTAAGATGTTAAATATATATACAAG 219
                   ||||||||||||||||||||||||||||||||||||||||||||||||||||||||||||
eccDNA Tos17:  131 TCTATTGCTAATACTATTGTTAGGTTGCAAGTTAGTTAAGATGTTAAATATATATACAAG 72

Read:          220 CTAATGTACTGTATAGTTGGCCCATGTCCAGCCCATCGGATGTCCAGCCCATTGGATCTT 279
                   ||||||||||||||||||||||||||||||||||||||||||||||| ||||||||||||
eccDNA Tos17:   71 CTAATGTACTGTATAGTTGGCCCATGTCCAGCCCATCGGATGTCCAGTCCATTGGATCTT 12

Read:          280 GTATCTTGTAT 290
                   |||||||||||
eccDNA Tos17:   11 GTATCTTGTAT 1
```

B

```
Read:              1  GGCCAACAGTAAAATAAGTCTAAGACTACATCGCTATACACATATCTAACATGTGAATTG  60
                      ||||||||||||||||||||||||||||||||||||||||||||||||||||||||||||
eccDNA Lullaby:  201  GGCCAACAGTAAAATAAGTCTAAGACTACATCGCTATACACATATCTAACATGTGAATTG  142

Read:             61  TGCTAAAATCAGTACGGTGTATTCCAGGAGAGCACTAGCATATACAATGGTATATGGGCC  120
                      ||||||||||||||||||||||||||||||||||||||||||||||||||||||||||||
eccDNA Lullaby:  141  TGCTAAAATCAGTACGGTGTATTCCAGGAGAGCACTAGCATATACAATGGTATATGGGCC  82
```

Supplementary Figure 8
