# Supplementary material for: Sequencing the extrachromosomal circular mobilome reveals retrotransposon activity in plants
Source: PLoS Genet. 2017 Feb 17;13(2):e1006630. doi: 10.1371/journal.pgen.1006630 (PMC5338827; doi:10.1371/journal.pgen.1006630)
Supplement: S10 Fig — (A) Example of a split read spanning the perfect junction of the 2LTR-circle corresponding to PopRice. (B) Example of a split read spanning the imperfect junction of the 2LTR-circle corresponding to PopRice. A primer binding site (PBS) sequence is highlighted in blue. The PBS is normally found after the 5’LTR in a linear PopRice. Legend as in S5 Fig. (PDF) [file pgen.1006630.s010.pdf]

A

```
Read:              2    CAACAAACTGTCCTAGAGCATCTCAACAAACTGCTGTCCTAACTGTCCTAGAGCATCCAA   61
                        ||||||||||||||||||||||||||||||||||||||||||||||||||||||||||||
eccDNA PopRice:  300    CAACAAACTGTCCTAGAGCATCTCAACAAACTGCTGTCCTAACTGTCCTAGAGCATCCAA   241

Read:             62    ACTGAAAGTAGAGATGCTGTCCTAGAAAGCTGAAAAGCAGGAAAAAGGCACCACAAGACC   121
                        ||||||||||||||||||||||||||||||||||||||||||||||||||||||||||||
eccDNA PopRice:  240    ACTGAAAGTAGAGATGCTGTCCTAGAAAGCTGAAAAGCAGGAAAAAGGCACCACAAGACC   181

Read:            122    AAGACCAACCAGCCAAGACTTATTCCTACATGTTAGAATTTCAATGGTTACTCTCATTGA   181
                        ||||||||||||||||||||||||||||||||||||||||||||||||||||||||||||
eccDNA PopRice:  180    AAGACCAACCAGCCAAGACTTATTCCTACATGTTAGAATTTCAATGGTTACTCTCATTGA   121

Read:            182    GAAGAGGATGGCACTCGAGATGGGGCAATTTTCTGGTTTTCTTCATTCACTAAACACAAA   241
                        ||||||||||||||||||||||||||||||||||||||||||||||||||||||||||||
eccDNA PopRice:  120    GAAGAGGATGGCACTCGAGATGGGGCAATTTTCTGGTTTTCTTCATTCACTAAACACAAA   61

Read:            242    AGCCATGTC   250
                        |||||||||
eccDNA PopRice:   60    AGCCATGTC   52
```

B

```
Read:              1    TCCAAACTGAAAGTAGAGATGCTGTCCTAGAAAGCTGAAAAGCAGGAAAAAGGCACCACA   60
                        ||||||||||||||||||||||||||||||||||||||||||||||||||||||||||||
eccDNA PopRice:  245    TCCAAACTGAAAGTAGAGATGCTGTCCTAGAAAGCTGAAAAGCAGGAAAAAGGCACCACA   186

Read:             61    AGACCAAGACCAACCAGCCAAGACTTATTCCTACAAGCCTACAGGAAAGTGTGGCTCTGA   120
                        |||||||||||||||||||||||||||||||||||
eccDNA PopRice:  185    AGACCAAGACCAACCAGCCAAGACTTATTCCTACA-------------------------   151

Read:            121    TACCAGATGTTAGAATTTCAATGGTTACTCTCATTGAGAAGAGGATGGCACTCGAGATGG   180
                               |||||||||||||||||||||||||||||||||||||||||||||||||||||
eccDNA PopRice:  150    -------TGTTAGAATTTCAATGGTTACTCTCATTGAGAAGAGGATGGCACTCGAGATGG   98

Read:            181    GGCAATTTTCTGGTTTTCTTCATTCACTAAACACAAAAGCCATGTCCTCCCAAAGAGGAT   240
                        ||||||||||||||||||||||||||||||||||||||||||||||||||||||||||||
eccDNA PopRice:   97    GGCAATTTTCTGGTTTTCTTCATTCACTAAACACAAAAGCCATGTCCTCCCAAAGAGGAT   38

Read:            241    TGGATACATAT   251
                        |||||||||||
eccDNA PopRice:   37    TGGATACATAT   27
```

Supplementary Figure 10
