# Supplementary material for: Sequencing the extrachromosomal circular mobilome reveals retrotransposon activity in plants
Source: PLoS Genet. 2017 Feb 17;13(2):e1006630. doi: 10.1371/journal.pgen.1006630 (PMC5338827; doi:10.1371/journal.pgen.1006630)
Supplement: S1 Table — (PDF) [file pgen.1006630.s014.pdf]

Supplementary Table 1. Characteristics of the *A. thaliana* mobilome-seq libraries.

Analysis \ Library	WT (At_WT)		epi12 (At_e12)	
	#1	#2	#1	#2
Library size (reads)	74,086	170,910	415,282	587,918
Reads mapping against organelles	18,288	39,808	161,924	151,128
Reads mapping against genome	31,373	69,160	132,390	208,653
Mean coverage per 100bp (rpm)	2.301	2.606	1.897	2.372
Total number of scaffolds (*de novo* assembly)	536	1548	863	2637
Number of *de novo* assembled scaffolds significantly covered ($p < 0.05$)	28	76	62	124
Mean coverage for split reads (segemehl mapping) per 100bp (rpm)	0.250	0.285	0.121	0.209
