# Supplementary material for: Sequencing the extrachromosomal circular mobilome reveals retrotransposon activity in plants
Source: PLoS Genet. 2017 Feb 17;13(2):e1006630. doi: 10.1371/journal.pgen.1006630 (PMC5338827; doi:10.1371/journal.pgen.1006630)
Supplement: S2 Table — (PDF) [file pgen.1006630.s015.pdf]

Supplementary Table 2. Characteristics of the *O. sativa* mobilome-seq libraries.

Analysis \ Library	Callus (Os_callus)			Leaf (Os_leaf)		Seed (Os_seed)	
	#1	#2	#3	#1	#2	#1	#2
Library size (reads)	1,941,638	4,012,812	1,715,560	2,948,984	2,754,020	2,183,780	1,313,588
Reads mapping against organelles	1,010,444	2,560,746	872,218	360,288	455,302	352,580	169,550
Reads mapping against genome	483,741	829,725	469,544	1,508,727	1,516,212	1,443,600	978,964
Mean coverage per 100bp (rpm)	0.707	0.662	0.692	0.648	0.614	0.599	0.675
Total number of scaffolds (*de novo* assembly)	1057	3377	849	10425	6558	7479	6506
Number of *de novo* assembled scaffolds significantly covered ($p < 0.05$)	85	200	62	325	237	423	316
Mean coverage for split reads (segemehl mapping) per 100bp (rpm)	0.030	0.043	0.037	0.076	0.13	0.058	0.040
