# Supplementary material for: Sequencing the extrachromosomal circular mobilome reveals retrotransposon activity in plants
Source: PLoS Genet. 2017 Feb 17;13(2):e1006630. doi: 10.1371/journal.pgen.1006630 (PMC5338827; doi:10.1371/journal.pgen.1006630)
Supplement: S3 Table — Nipponbare reference genome. (PDF) [file pgen.1006630.s016.pdf]

Supplementary Table 3. Localization of *PopRice* and *osr4* elements in the *O. sativa ssp. japonica* cv. Nipponbare reference genome.

Name	Chromosome	Start	End
PopRice_1	chr01	4 776 239	4 781 940
osr4_10	chr01	9 619 047	9 624 779
osr4_1	chr02	3 433 922	3 439 683
osr4_30	chr02	4 810 186	4 815 148
osr4_28	chr02	6 298 856	6 304 425
osr4_23	chr02	10 230 034	10 235 735
PopRice_2	chr02	11 897 051	11 902 751
osr4_11	chr02	17 177 630	17 183 380
osr4_29	chr02	20 017 590	20 022 447
osr4_24	chr02	32 073 359	32 079 087
PopRice_3	chr02	34 010 137	34 015 809
osr4_12	chr03	1 805 958	1 811 710
PopRice_4	chr03	1 910 824	1 916 518
osr4_2	chr03	9 807 146	9 812 816
osr4_22	chr04	11 784 416	11 790 165
PopRice_5	chr04	21 858 331	21 863 990
osr4_19	chr04	24 624 991	24 630 730
PopRice_6	chr04	29 764 342	29 769 992
PopRice_7	chr04	31 205 979	31 211 679
osr4_3	chr05	18 149 395	18 155 117
osr4_8	chr05	18 173 196	18 178 845
PopRice_8	chr06	2 568 077	2 573 729
PopRice_9	chr06	3 207 031	3 212 695
osr4_18	chr06	13 707 057	13 712 806
osr4_21	chr06	24 968 651	24 974 392
osr4_27	chr07	10 183 108	10 188 828
PopRice_10	chr07	11 227 404	11 233 065
PopRice_11	chr08	9 051 840	9 057 543
osr4_25	chr08	14 745 774	14 751 442
osr4_5	chr08	25 668 348	25 674 136
PopRice_12	chr09	1 229 148	1 234 789
osr4_9	chr09	6 996 940	7 001 178
PopRice_13	chr09	8 572 145	8 577 847
osr4_6	chr09	18 114 586	18 120 338
osr4_13	chr09	19 370 956	19 376 699
PopRice_14	chr10	13 174 222	13 179 845
osr4_20	chr10	18 476 622	18 482 493
PopRice_15	chr10	22 300 481	22 306 180
osr4_14	chr10	22 402 727	22 408 469
PopRice_16	chr11	4 997 937	5 003 672
PopRice_17	chr11	26 689 013	26 694 658
osr4_17	chr12	4 851 245	4 856 938
osr4_16	chr12	5 895 106	5 900 860
osr4_4	chr12	11 047 463	11 053 165
osr4_26	chr12	21 273 154	21 278 677
osr4_7	chr12	23 149 361	23 155 098
osr4_15	chr12	24 767 171	24 772 919
