# Supplementary material for: Sequencing the extrachromosomal circular mobilome reveals retrotransposon activity in plants
Source: PLoS Genet. 2017 Feb 17;13(2):e1006630. doi: 10.1371/journal.pgen.1006630 (PMC5338827; doi:10.1371/journal.pgen.1006630)
Supplement: S4 Table — (PDF) [file pgen.1006630.s017.pdf]

Supplementary Table 4. List of primers.

**Assay**	**Primers name**	**Forward sequence (5'-3')**	**Reverse sequence (5'-3')**	**Amplicon size (bp)**	**Tm (°C)**
EVD probe (Southern blot)	Ty1_1_F/ Ty1_406_R	TATTGATCAAGACTCAAATAAGAAAGG	TGAAAGAATATGCGGAATTGTATTAAA	406	55
eEF1α (O. sativa)	2460F/2749R	GATCTGGTAAGGAGCTGGAGAAGG	CCGTGCACAAAACTACCACTTGAA	313	58
Tos17 (linear)	19F/635R	CTAATGTACTGTATAGTTGGCCC	ACCAAAGATCACTAGCAACTC	581	60
Tos17 (circular)	SL4F/SL4R	AACTCGAGAGCATCATCGGTTACA	CATTAGCTGTATGAACGGTGGCAC	1570-1428	62
PopRice (linear)	514F/799R	CTCGTTAGCTGGCAGTCAATCAAG	CTTCCTCCAAGTAGCTTCGGATGA	285	60
PopRice (circular)	26F/283R	ACAAACTGCTGTCCTAACTGTCCT	GCAGCTATAAATATGTATCCAATCCT	258	55
PopRice RT-qPCR	SL32F/SL32R	GAGGACGACGTTATTTCCTG	GAGGACCATCACCCACATGTA	65	60
PopRice probe (Southern blot)	RT1F/RT1R	GTTATTTCCTGCTGCTCGTCGAC	GTCGCCGAGAAGATCCTCCATC	1022	57
Osr4 RT-qPCR	SL33F/SL33R	TTGCTCGACTGCTTAGTGAT	GGACTTGCTATCCACCCTGA	71	60
