# Supplementary material for: Sequencing the extrachromosomal circular mobilome reveals retrotransposon activity in plants
Source: PLoS Genet. 2017 Feb 17;13(2):e1006630. doi: 10.1371/journal.pgen.1006630 (PMC5338827; doi:10.1371/journal.pgen.1006630)
Supplement: S5 Table — For each library, the number of mapped reads per million per 100bp window is indicated with P-value < 10−3 (CHR: chromosome, BP: base pair start coordinate of the 100bp window). (PDF) [file pgen.1006630.s018.pdf]

Supplementary Table 5. Table with significant coverage values for 100 bp windows. For each library, the number of mapped reads per million per 100bp window is indicated with P-value < 10^{-3} (CHR: chromosome, BP: base pair start coordinate of the 100bp window).

CHR	BP	TE family	Reads per million reads	P-value	Mobilome library
1	13955100	ATLANTYS1	2024	1.37E-06	*A. thaliana* WT
1	13955200	ATLANTYS1	2415	7.85E-08	*A. thaliana* WT
1	13955300	ATLANTYS1	1894	3.55E-06	*A. thaliana* WT
1	13955400	ATLANTYS1	1403	1.25E-04	*A. thaliana* WT
2	16045500	ATDNAI27T9A	2400	8.73E-08	*A. thaliana* WT
2	16045600	ATDNAI27T9A	2487	4.61E-08	*A. thaliana* WT
3	14251300	ATLANTYS3	1171	6.49E-04	*A. thaliana* WT
4	2915300	ATLANTYS1	1345	1.89E-04	*A. thaliana* WT
4	3625500	ATLANTYS1	1576	3.57E-05	*A. thaliana* WT
4	3625600	ATLANTYS1	1721	1.25E-05	*A. thaliana* WT
4	3625700	ATLANTYS1	1460	8.22E-05	*A. thaliana* WT
5	13182100	ATMU1	1128	8.83E-04	*A. thaliana* WT
1	12362700	ATREP13	1548	4.09E-04	*A. thaliana* epi12
1	12363000	ATREP13	2641	2.15E-06	*A. thaliana* epi12
1	12363100	ATREP13	3053	2.99E-07	*A. thaliana* epi12
1	12754300	ATCOPIA93	1850	9.56E-05	*A. thaliana* epi12
1	12754400	ATCOPIA93	3532	3.03E-08	*A. thaliana* epi12
1	12754500	ATCOPIA93	3245	1.20E-07	*A. thaliana* epi12
1	12754600	ATCOPIA93	2152	2.24E-05	*A. thaliana* epi12
1	12754700	ATCOPIA93	1558	3.91E-04	*A. thaliana* epi12
1	12754900	ATCOPIA93	2909	5.95E-07	*A. thaliana* epi12
1	12755000	ATCOPIA93	7553	2.22E-16	*A. thaliana* epi12
1	12755100	ATCOPIA93	12188	0.00E+00	*A. thaliana* epi12
1	12755200	ATCOPIA93	13146	0.00E+00	*A. thaliana* epi12
1	12755300	ATCOPIA93	10366	0.00E+00	*A. thaliana* epi12
1	12755400	ATCOPIA93	4467	3.51E-10	*A. thaliana* epi12
1	12758400	ATCOPIA93	1701	1.95E-04	*A. thaliana* epi12
1	12758500	ATCOPIA93	2233	1.51E-05	*A. thaliana* epi12
1	12758600	ATCOPIA93	2454	5.26E-06	*A. thaliana* epi12
1	12758700	ATCOPIA93	1845	9.78E-05	*A. thaliana* epi12
1	12758800	ATCOPIA93	1716	1.82E-04	*A. thaliana* epi12
1	12758900	ATCOPIA93	1577	3.56E-04	*A. thaliana* epi12
1	12759000	ATCOPIA93	1505	5.04E-04	*A. thaliana* epi12
1	12759600	ATCOPIA93	1606	3.10E-04	*A. thaliana* epi12
1	12759700	ATCOPIA93	1395	8.58E-04	*A. thaliana* epi12
1	12760000	ATCOPIA93	5799	6.15E-13	*A. thaliana* epi12
1	12760100	ATCOPIA93	11196	0.00E+00	*A. thaliana* epi12
1	12760200	ATCOPIA93	11018	0.00E+00	*A. thaliana* epi12
1	12760300	ATCOPIA93	5799	6.15E-13	*A. thaliana* epi12
1	16583000	ATENSPM9	2003	4.57E-05	*A. thaliana* epi12
1	16583100	ATENSPM9	1965	5.50E-05	*A. thaliana* epi12
2	5829200	ATGP5	2785	1.08E-06	*A. thaliana* epi12
2	5829300	ATGP5	3125	2.12E-07	*A. thaliana* epi12
3	10181900	ATCOPIA93	3642	1.79E-08	*A. thaliana* epi12
3	10182000	ATCOPIA93	7659	1.11E-16	*A. thaliana* epi12
3	10182100	ATCOPIA93	11603	0.00E+00	*A. thaliana* epi12
3	10182200	ATCOPIA93	12077	0.00E+00	*A. thaliana* epi12
3	10182300	ATCOPIA93	7376	3.33E-16	*A. thaliana* epi12
3	11357300	ATCOPIA41	1759	1.48E-04	*A. thaliana* epi12

CHR	BP	TE family	Reads per million reads	P-value	Mobilome library
3	11357400	ATCOPIA41	3130	2.07E-07	*A. thaliana* epi12
3	11357500	ATCOPIA41	3192	1.54E-07	*A. thaliana* epi12
3	11357600	ATCOPIA41	4007	3.15E-09	*A. thaliana* epi12
3	11357700	ATCOPIA41	2722	1.45E-06	*A. thaliana* epi12
3	11357800	ATCOPIA41	2051	3.63E-05	*A. thaliana* epi12
4	4594400	ATLINE2	1778	1.35E-04	*A. thaliana* epi12
4	4594500	ATLINE2	1778	1.35E-04	*A. thaliana* epi12
5	5630000	ATCOPIA93	6240	7.55E-14	*A. thaliana* epi12
5	5630100	ATCOPIA93	13232	0.00E+00	*A. thaliana* epi12
5	5630200	ATCOPIA93	17263	0.00E+00	*A. thaliana* epi12
5	5630300	ATCOPIA93	19827	0.00E+00	*A. thaliana* epi12
5	5630400	ATCOPIA93	19966	0.00E+00	*A. thaliana* epi12
5	5630500	ATCOPIA93	19875	0.00E+00	*A. thaliana* epi12
5	5630600	ATCOPIA93	15384	0.00E+00	*A. thaliana* epi12
5	5630700	ATCOPIA93	13213	0.00E+00	*A. thaliana* epi12
5	5630800	ATCOPIA93	9988	0.00E+00	*A. thaliana* epi12
5	5630900	ATCOPIA93	8171	0.00E+00	*A. thaliana* epi12
5	5631000	ATCOPIA93	8200	0.00E+00	*A. thaliana* epi12
5	5631100	ATCOPIA93	6259	6.88E-14	*A. thaliana* epi12
5	5631200	ATCOPIA93	5281	7.23E-12	*A. thaliana* epi12
5	5631300	ATCOPIA93	4031	2.81E-09	*A. thaliana* epi12
5	5631400	ATCOPIA93	3930	4.53E-09	*A. thaliana* epi12
5	5631500	ATCOPIA93	3738	1.13E-08	*A. thaliana* epi12
5	5631600	ATCOPIA93	3139	1.98E-07	*A. thaliana* epi12
5	5631700	ATCOPIA93	2756	1.24E-06	*A. thaliana* epi12
5	5631800	ATCOPIA93	1749	1.55E-04	*A. thaliana* epi12
5	5631900	ATCOPIA93	1510	4.92E-04	*A. thaliana* epi12
5	5632900	ATCOPIA93	1802	1.20E-04	*A. thaliana* epi12
5	5633000	ATCOPIA93	1677	2.19E-04	*A. thaliana* epi12
5	5633100	ATCOPIA93	1740	1.62E-04	*A. thaliana* epi12
5	5633200	ATCOPIA93	1735	1.66E-04	*A. thaliana* epi12
5	5633300	ATCOPIA93	2181	1.95E-05	*A. thaliana* epi12
5	5633400	ATCOPIA93	2382	7.43E-06	*A. thaliana* epi12
5	5633500	ATCOPIA93	2368	7.96E-06	*A. thaliana* epi12
5	5633600	ATCOPIA93	2003	4.57E-05	*A. thaliana* epi12
5	5633700	ATCOPIA93	1682	2.14E-04	*A. thaliana* epi12
5	5633800	ATCOPIA93	1764	1.45E-04	*A. thaliana* epi12
5	5633900	ATCOPIA93	3158	1.81E-07	*A. thaliana* epi12
5	5634000	ATCOPIA93	4529	2.61E-10	*A. thaliana* epi12
5	5634100	ATCOPIA93	5837	5.12E-13	*A. thaliana* epi12
5	5634200	ATCOPIA93	8526	0.00E+00	*A. thaliana* epi12
5	5634300	ATCOPIA93	11833	0.00E+00	*A. thaliana* epi12
5	5634400	ATCOPIA93	14747	0.00E+00	*A. thaliana* epi12
5	5634500	ATCOPIA93	19175	0.00E+00	*A. thaliana* epi12
5	5634600	ATCOPIA93	18389	0.00E+00	*A. thaliana* epi12
5	5634700	ATCOPIA93	21361	0.00E+00	*A. thaliana* epi12
5	5634800	ATCOPIA93	18965	0.00E+00	*A. thaliana* epi12
5	5634900	ATCOPIA93	16798	0.00E+00	*A. thaliana* epi12
5	5635000	ATCOPIA93	13836	0.00E+00	*A. thaliana* epi12

CHR	BP	TE family	Reads per million reads	P-value	Mobilome library
5	5635100	ATCOPIA93	11037	0.00E+00	*A. thaliana* epi12
5	5635200	ATCOPIA93	6365	4.17E-14	*A. thaliana* epi12
7	26695100	LTR_fam158_tos17_expop1+	480	7.84E-04	*O. sativa* callus
7	26695200	LTR_fam158_tos17_expop1+	610	1.31E-04	*O. sativa* callus
7	26695300	LTR_fam158_tos17_expop1+	575	2.12E-04	*O. sativa* callus
7	26695400	LTR_fam158_tos17_expop1+	587	1.79E-04	*O. sativa* callus
7	26697900	LTR_fam158_tos17_expop1+	505	5.53E-04	*O. sativa* callus
7	26698000	LTR_fam158_tos17_expop1+	632	9.74E-05	*O. sativa* callus
7	26698100	LTR_fam158_tos17_expop1+	670	5.75E-05	*O. sativa* callus
7	26698200	LTR_fam158_tos17_expop1+	629	1.01E-04	*O. sativa* callus
7	26698300	LTR_fam158_tos17_expop1+	478	7.97E-04	*O. sativa* callus
7	26698500	LTR_fam158_tos17_expop1+	491	6.75E-04	*O. sativa* callus
7	26698600	LTR_fam158_tos17_expop1+	538	3.54E-04	*O. sativa* callus
7	26698700	LTR_fam158_tos17_expop1+	645	8.12E-05	*O. sativa* callus
7	26698800	LTR_fam158_tos17_expop1+	463	9.90E-04	*O. sativa* callus
1	4776300	LTR_fam51_osr4_poprice_expop1+	46	8.97E-05	*O. sativa* seed
1	4776400	LTR_fam51_osr4_poprice_expop1+	77	9.05E-09	*O. sativa* seed
1	4776500	LTR_fam51_osr4_poprice_expop1+	97	1.42E-11	*O. sativa* seed
1	4776600	LTR_fam51_osr4_poprice_expop1+	83	1.33E-09	*O. sativa* seed
1	4776700	LTR_fam51_osr4_poprice_expop1+	40	5.01E-04	*O. sativa* seed
1	4781700	LTR_fam51_osr4_poprice_expop1+	60	1.35E-06	*O. sativa* seed
1	4781800	LTR_fam51_osr4_poprice_expop1+	54	8.38E-06	*O. sativa* seed
1	4781900	LTR_fam51_osr4_poprice_expop1+	41	3.78E-04	*O. sativa* seed
1	28292600	DTM_MULE_japo_Os0086	39	6.64E-04	*O. sativa* seed
1	28292700	DTM_MULE_japo_Os0086	260	0.00E+00	*O. sativa* seed
1	28292800	DTM_MULE_japo_Os0086	266	0.00E+00	*O. sativa* seed
1	28292900	DTM_MULE_japo_Os0086	48	5.00E-05	*O. sativa* seed
2	11897000	LTR_fam51_osr4_poprice_expop1+	46	8.97E-05	*O. sativa* seed
2	11897100	LTR_fam51_osr4_poprice_expop1+	93	5.22E-11	*O. sativa* seed
2	11897200	LTR_fam51_osr4_poprice_expop1+	116	2.66E-14	*O. sativa* seed
2	11897300	LTR_fam51_osr4_poprice_expop1+	103	1.97E-12	*O. sativa* seed
2	11897400	LTR_fam51_osr4_poprice_expop1+	81	2.52E-09	*O. sativa* seed
2	11897500	LTR_fam51_osr4_poprice_expop1+	48	5.00E-05	*O. sativa* seed
2	11902200	LTR_fam51_osr4_poprice_expop1+	46	8.97E-05	*O. sativa* seed
2	11902300	LTR_fam51_osr4_poprice_expop1+	126	1.33E-15	*O. sativa* seed
2	11902400	LTR_fam51_osr4_poprice_expop1+	156	0.00E+00	*O. sativa* seed
2	11902500	LTR_fam51_osr4_poprice_expop1+	162	0.00E+00	*O. sativa* seed
2	11902600	LTR_fam51_osr4_poprice_expop1+	100	5.29E-12	*O. sativa* seed
2	11902700	LTR_fam51_osr4_poprice_expop1+	50	2.77E-05	*O. sativa* seed
2	33615400	DTM_clust113	72	4.41E-08	*O. sativa* seed
2	33615500	DTM_clust113	48	5.00E-05	*O. sativa* seed
2	33658200	rn_118-68_expop1+	41	3.78E-04	*O. sativa* seed
2	34010100	LTR_fam51_osr4_poprice_expop1+	70	6.04E-08	*O. sativa* seed
2	34010200	LTR_fam51_osr4_poprice_expop1+	122	5.00E-15	*O. sativa* seed
2	34010300	LTR_fam51_osr4_poprice_expop1+	159	0.00E+00	*O. sativa* seed
2	34010400	LTR_fam51_osr4_poprice_expop1+	139	0.00E+00	*O. sativa* seed
2	34010500	LTR_fam51_osr4_poprice_expop1+	83	1.33E-09	*O. sativa* seed
2	34010600	LTR_fam51_osr4_poprice_expop1+	48	5.00E-05	*O. sativa* seed
2	34015300	LTR_fam51_osr4_poprice_expop1+	60	1.35E-06	*O. sativa* seed

CHR	BP	TE family	Reads per million reads	P-value	Mobilome library
**2**	34015400	LTR_fam51_osr4_poprice_expop1+	129	4.44E-16	*O. sativa* seed
**2**	34015500	LTR_fam51_osr4_poprice_expop1+	157	0.00E+00	*O. sativa* seed
**2**	34015600	LTR_fam51_osr4_poprice_expop1+	160	0.00E+00	*O. sativa* seed
**2**	34015700	LTR_fam51_osr4_poprice_expop1+	91	1.00E-10	*O. sativa* seed
**3**	1910900	LTR_fam51_osr4_poprice_expop1+	43	2.14E-04	*O. sativa* seed
**3**	1911000	LTR_fam51_osr4_poprice_expop1+	40	5.01E-04	*O. sativa* seed
**3**	1916200	LTR_fam51_osr4_poprice_expop1+	46	8.97E-05	*O. sativa* seed
**3**	1916300	LTR_fam51_osr4_poprice_expop1+	57	3.38E-06	*O. sativa* seed
**3**	1916400	LTR_fam51_osr4_poprice_expop1+	40	5.01E-04	*O. sativa* seed
**4**	11804800	LTR_fam51_osr4_poprice_expop1+	42	2.84E-04	*O. sativa* seed
**4**	21858400	LTR_fam51_osr4_poprice_expop1+	59	1.84E-06	*O. sativa* seed
**4**	21858500	LTR_fam51_osr4_poprice_expop1+	64	3.93E-07	*O. sativa* seed
**4**	21863500	LTR_fam51_osr4_poprice_expop1+	44	1.60E-04	*O. sativa* seed
**4**	21863600	LTR_fam51_osr4_poprice_expop1+	51	2.06E-05	*O. sativa* seed
**4**	21863700	LTR_fam51_osr4_poprice_expop1+	59	1.84E-06	*O. sativa* seed
**4**	21863800	LTR_fam51_osr4_poprice_expop1+	54	8.38E-06	*O. sativa* seed
**4**	31206000	LTR_fam51_osr4_poprice_expop1+	63	5.36E-07	*O. sativa* seed
**4**	31206100	LTR_fam51_osr4_poprice_expop1+	112	1.01E-13	*O. sativa* seed
**4**	31206200	LTR_fam51_osr4_poprice_expop1+	112	1.01E-13	*O. sativa* seed
**4**	31206300	LTR_fam51_osr4_poprice_expop1+	83	1.33E-09	*O. sativa* seed
**4**	31210900	LTR_fam51_osr4_poprice_expop1+	44	1.60E-04	*O. sativa* seed
**4**	31211000	LTR_fam51_osr4_poprice_expop1+	48	5.00E-05	*O. sativa* seed
**4**	31211100	LTR_fam51_osr4_poprice_expop1+	48	5.00E-05	*O. sativa* seed
**4**	31211200	LTR_fam51_osr4_poprice_expop1+	66	2.11E-07	*O. sativa* seed
**4**	31211300	LTR_fam51_osr4_poprice_expop1+	107	5.28E-13	*O. sativa* seed
**4**	31211400	LTR_fam51_osr4_poprice_expop1+	151	0.00E+00	*O. sativa* seed
**4**	31211500	LTR_fam51_osr4_poprice_expop1+	89	1.92E-10	*O. sativa* seed
**4**	31211600	LTR_fam51_osr4_poprice_expop1+	54	8.38E-06	*O. sativa* seed
**5**	27400400	DTM_MULE_japo_Os0314.DTM_clust416	39	6.64E-04	*O. sativa* seed
**6**	28571500	GAIJIN_DNA_transposon_Oryza_sativa	41	3.78E-04	*O. sativa* seed
**6**	28571600	DTX-incomp-chim_Osati-B-R2026-Map10.GAIJIN_DNA_transposon_Oryza_sativa.RSU_clust154	68	1.13E-07	*O. sativa* seed
**7**	26694800	LTR_fam158_tos17_expop1+	54	8.38E-06	*O. sativa* seed
**7**	26694900	LTR_fam158_tos17_expop1+	53	1.13E-05	*O. sativa* seed
**7**	26695000	LTR_fam158_tos17_expop1+	51	2.06E-05	*O. sativa* seed
**7**	26695100	LTR_fam158_tos17_expop1+	44	1.60E-04	*O. sativa* seed
**7**	26695200	LTR_fam158_tos17_expop1+	53	1.13E-05	*O. sativa* seed
**7**	26695300	LTR_fam158_tos17_expop1+	48	5.00E-05	*O. sativa* seed
**7**	26695400	LTR_fam158_tos17_expop1+	46	8.97E-05	*O. sativa* seed
**7**	26698700	LTR_fam158_tos17_expop1+	43	2.14E-04	*O. sativa* seed
**8**	9051800	DHX-incomp_Osati-B-R11497-Map7_reversed.LTR_fam51_osr4_poprice_expop1+	42	2.84E-04	*O. sativa* seed
**8**	9051900	LTR_fam51_osr4_poprice_expop1+	92	7.23E-11	*O. sativa* seed
**8**	9052000	LTR_fam51_osr4_poprice_expop1+	113	7.22E-14	*O. sativa* seed
**8**	9052100	LTR_fam51_osr4_poprice_expop1+	86	5.06E-10	*O. sativa* seed
**8**	9052200	LTR_fam51_osr4_poprice_expop1+	54	8.38E-06	*O. sativa* seed
**8**	9057000	LTR_fam51_osr4_poprice_expop1+	38	8.77E-04	*O. sativa* seed
**8**	9057100	LTR_fam51_osr4_poprice_expop1+	52	1.53E-05	*O. sativa* seed
**8**	9057200	LTR_fam51_osr4_poprice_expop1+	61	9.94E-07	*O. sativa* seed
**8**	9057300	LTR_fam51_osr4_poprice_expop1+	67	1.55E-07	*O. sativa* seed
**8**	9057400	LTR_fam51_osr4_poprice_expop1+	53	1.13E-05	*O. sativa* seed

CHR	BP	TE family	Reads per million reads	P-value	Mobilome library
**8**	15220800	LTR_fam86_expop1+	42	2.84E-04	*O.sativa* seed
**8**	25668500	LTR_fam51_osr4_poprice_expop1+	48	5.00E-05	*O.sativa* seed
**8**	25668600	LTR_fam51_osr4_poprice_expop1+	38	8.77E-04	*O.sativa* seed
**8**	25668700	LTR_fam51_osr4_poprice_expop1+	41	3.78E-04	*O.sativa* seed
**8**	25668800	LTR_fam51_osr4_poprice_expop1+	40	5.01E-04	*O.sativa* seed
**9**	1229300	LTR_fam51_osr4_poprice_expop1+	45	1.20E-04	*O.sativa* seed
**9**	8362200	LTR_fam29_rire10_expop1+	46	8.97E-05	*O.sativa* seed
**9**	8362300	LTR_fam29_rire10_expop1+	63	5.36E-07	*O.sativa* seed
**9**	8362400	LTR_fam29_rire10_expop1+	51	2.06E-05	*O.sativa* seed
**9**	8572300	LTR_fam51_osr4_poprice_expop1+	46	8.97E-05	*O.sativa* seed
**9**	8577500	LTR_fam51_osr4_poprice_expop1+	48	5.00E-05	*O.sativa* seed
**9**	18114700	LTR_fam51_osr4_poprice_expop1+	38	8.77E-04	*O.sativa* seed
**9**	18114800	LTR_fam51_osr4_poprice_expop1+	46	8.97E-05	*O.sativa* seed
**9**	18114900	LTR_fam51_osr4_poprice_expop1+	44	1.60E-04	*O.sativa* seed
**9**	18119400	LTR_fam51_osr4_poprice_expop1+	39	6.64E-04	*O.sativa* seed
**9**	18119500	LTR_fam51_osr4_poprice_expop1+	55	6.20E-06	*O.sativa* seed
**9**	18119600	LTR_fam51_osr4_poprice_expop1+	94	3.77E-11	*O.sativa* seed
**9**	18119700	LTR_fam51_osr4_poprice_expop1+	110	1.95E-13	*O.sativa* seed
**9**	18119800	LTR_fam51_osr4_poprice_expop1+	140	0.00E+00	*O.sativa* seed
**9**	18119900	LTR_fam51_osr4_poprice_expop1+	133	1.11E-16	*O.sativa* seed
**9**	18120000	LTR_fam51_osr4_poprice_expop1+	106	7.34E-13	*O.sativa* seed
**9**	18120100	LTR_fam51_osr4_poprice_expop1+	80	3.47E-09	*O.sativa* seed
**9**	18120200	LTR_fam51_osr4_poprice_expop1+	48	5.00E-05	*O.sativa* seed
**10**	2994100	DTM_MULE_japo_Os3337	42	2.84E-04	*O.sativa* seed
**10**	2994200	DTM_MULE_japo_Os3337	59	1.84E-06	*O.sativa* seed
**10**	2994300	DTM_MULE_japo_Os3337	65	2.88E-07	*O.sativa* seed
**10**	22300600	LTR_fam51_osr4_poprice_expop1+	70	6.04E-08	*O.sativa* seed
**10**	22300700	LTR_fam51_osr4_poprice_expop1+	76	1.24E-08	*O.sativa* seed
**10**	22300800	LTR_fam51_osr4_poprice_expop1+	67	1.55E-07	*O.sativa* seed
**10**	22300900	LTR_fam51_osr4_poprice_expop1+	46	8.97E-05	*O.sativa* seed
**10**	22305900	LTR_fam51_osr4_poprice_expop1+	55	6.20E-06	*O.sativa* seed
**10**	22306000	LTR_fam51_osr4_poprice_expop1+	54	8.38E-06	*O.sativa* seed
**11**	8983500	DTX-incomp-chim_Osati-B-G3264-Map20.LTR_fam73_expop1+	39	6.64E-04	*O.sativa* seed
**12**	673100	LTR_fam4_dasheng_osr25_expop1+	40	5.01E-04	*O.sativa* seed
