# Supplementary material for: Sequencing the extrachromosomal circular mobilome reveals retrotransposon activity in plants
Source: PLoS Genet. 2017 Feb 17;13(2):e1006630. doi: 10.1371/journal.pgen.1006630 (PMC5338827; doi:10.1371/journal.pgen.1006630)
Supplement: S6 Table — For each library, the peaks corresponding to candidate TEs are listed. (PDF) [file pgen.1006630.s019.pdf]

library	chr	start	end	family	length (bp)	DOC coverage (rpm)	SR coverage (rpm)
At_WT_1	Chr1	16093188	16093881	ATHILA4C	693	229	54
At_WT_1	Chr5	14296910	14297141	ATHILA4	231	148	67
At_WT_1	Chr2	2346315	2346491	RP1_AT	176	121	162
At_WT_1	Chr1	5357353	5357511	ATTIRTA1	158	108	94
At_WT_2	Chr2	16045588	16045689	ATDNAI27T9A	101	1001	53
At_WT_2	Chr1	16093188	16093881	ATHILA4C	693	252	53
At_WT_2	Chr2	3481566	3482049	TA11	483	193	322
At_WT_2	Chr2	3275023	3275258	ATCOPIA50	235	88	47
At_WT_2	Chr2	2346315	2346491	RP1_AT	176	64	29
At_WT_2	Chr2	19629360	19629469	AT9TSD1	109	64	53
At_WT_2	Chr5	1115681	1115832	ATLINE1_4	151	59	18
At_WT_2	Chr2	4022986	4023317	ATREP3	331	53	23
At_WT_2	Chr2	4430013	4430630	VANDAL4	617	47	23
At_WT_2	Chr2	3440471	3440652	ATCOPIA85	181	47	29
At_WT_2	Chr1	5357353	5357511	ATTIRTA1	158	47	47
At_WT_2	Chr5	14560838	14561196	ATDNA12T3_2	358	41	29
At_WT_2	Chr3	14263320	14263420	ATHILA5	100	35	18
At_WT_2	Chr3	12700743	12700878	ATHILA4A	135	35	18
At_WT_2	Chr5	1655270	1655409	ATREP10A	139	23	18
At_e12_1	Chr5	5629977	5635310	ATCOPIA93	5 333	20331	1406
At_e12_1	Chr1	12755059	12760395	ATCOPIA93	5 336	7946	1192
At_e12_1	Chr1	12753511	12755059	ATCOPIA93	1 548	2345	284
At_e12_1	Chr1	12362546	12363127	ATREP13	581	614	104
At_e12_1	Chr3	12053387	12054530	ATHILA2	1 143	532	41
At_e12_1	Chr1	27932605	27933669	TAG2	1 064	366	41
At_e12_1	Chr2	1297909	1298511	ATENSPM5	602	255	14
At_e12_1	Chr3	11678189	11678764	ATCOPIA95	575	205	12
At_e12_1	Chr3	17914130	17914576	ATCOPIA73	446	111	17
At_e12_1	Chr1	30005633	30006120	TAG1	487	96	22
At_e12_1	Chr1	24275018	24275588	ATCOPIA15	570	48	41
At_e12_1	Chr2	3318352	3318625	TA11	273	34	14
At_e12_1	Chr2	3275023	3275258	ATCOPIA50	235	31	24
At_e12_1	Chr2	3344834	3345276	ATCOPIA66	442	12	14
At_e12_2	Chr5	5629977	5635310	ATCOPIA93	5 333	48985	7639
At_e12_2	Chr1	12755059	12760395	ATCOPIA93	5 336	15509	5171
At_e12_2	Chr1	12753511	12755059	ATCOPIA93	1 548	4730	1155
At_e12_2	Chr1	12362546	12363127	ATREP13	581	1941	321
At_e12_2	Chr3	12053387	12054530	ATHILA2	1 143	236	12
At_e12_2	Chr2	1297909	1298511	ATENSPM5	602	184	20
At_e12_2	Chr3	18833231	18833806	ATLINEIII	575	184	66
At_e12_2	Chr2	6513788	6514170	ATLINE1_2	382	124	19
At_e12_2	Chr2	3275023	3275258	ATCOPIA50	235	117	26
At_e12_2	Chr4	4609216	4610745	ATHILA0_I	1 529	109	34
At_e12_2	Chr4	4948153	4949024	ATREP10B	871	97	10
At_e12_2	Chr3	15172904	15173473	ATHATN7	569	95	12
At_e12_2	Chr2	3379193	3379763	ATCOPIA15	570	92	12
At_e12_2	Chr1	24275018	24275588	ATCOPIA15	570	83	31
At_e12_2	Chr2	3282018	3282135	ATCOPIA50	117	77	15
At_e12_2	Chr1	30005633	30006120	TAG1	487	68	12
At_e12_2	Chr3	15250438	15250952	ATHILA2	514	65	10
At_e12_2	Chr4	3336731	3337334	TAT1_ATH	603	58	22
At_e12_2	Chr3	11678189	11678764	ATCOPIA95	575	51	17
At_e12_2	Chr4	7990413	7990601	ATREP4	188	48	17
At_e12_2	Chr2	3440471	3440652	ATCOPIA85	181	39	26
At_e12_2	Chr5	9487451	9488211	ATREP1	760	37	19
At_e12_2	Chr4	3054741	3055303	ATDNA12T3_2	562	34	12
At_e12_2	Chr1	11782132	11782287	ATREP11	155	31	17
At_e12_2	Chr4	4467119	4467241	ATMU2	122	22	19
At_e12_2	Chr2	5950639	5950819	HELITRONY3	180	19	17
At_e12_2	Chr4	850521	850663	ATLINE1A	142	17	10
At_e12_2	Chr3	9394480	9394588	HELITRONY1D	108	17	14
At_e12_2	Chr2	3344834	3345276	ATCOPIA66	442	15	10
At_e12_2	Chr1	25344308	25344450	ATHATN2	142	14	15
Os_callus_1	chr07	26695361	26698920	LTR_fam158_tos17	3559	1140	174
Os_callus_1	chr10	15415372	15419577	LTR_fam158_tos17	4205	741	97
Os_callus_1	chr07	26694787	26695362	LTR_fam158_tos17	575	189	80
Os_callus_1	chr08	25347504	25347753	LTR_fam149_rn_161-163	249	17	27
Os_callus_1	chr08	25347480	25347638	rn_267-152	158	16	18
Os_callus_2	chr07	26695361	26698920	LTR_fam158_tos17	3559	863	140
Os_callus_2	chr10	15415372	15419577	LTR_fam158_tos17	4205	589	61
Os_callus_2	chr07	26694787	26695362	LTR_fam158_tos17	575	160	57
Os_callus_2	chr08	25347504	25347753	LTR_fam149_rn_161-163	249	20	12
Os_seed_1	chr02	34010327	34015744	LTR_fam51_osr4	5417	375	144
Os_seed_1	chr02	11897228	11902686	LTR_fam51_osr4	5458	347	196

Os_seed_1	chr04	31206043	31211501	LTR_fam51_osr4	5458	336	128
Os_seed_1	chr01	4776416	4781875	LTR_fam51_osr4	5459	216	52
Os_seed_1	chr07	26695361	26698920	LTR_fam158_tos17	3559	161	97
Os_seed_1	chr04	31211382	31211739	LTR_fam51_osr4	357	132	14
Os_seed_1	chr02	34010136	34010357	LTR_fam51_osr4	221	123	66
Os_seed_1	chr02	34015614	34015809	LTR_fam51_osr4	195	119	33
Os_seed_1	chr02	11897047	11897347	LTR_fam51_osr4	300	118	39
Os_seed_1	chr09	18119942	18120325	LTR_fam51_osr4	383	114	14
Os_seed_1	chr02	11902556	11902751	LTR_fam51_osr4	195	109	91
Os_seed_1	chr02	11897050	11897281	LTR_fam51_osr4	231	94	33
Os_seed_1	chr08	9051839	9052035	LTR_fam51_osr4	196	83	27
Os_seed_1	chr04	31205978	31206173	LTR_fam51_osr4	195	82	81
Os_seed_1	chr10	22300480	22300791	LTR_fam51_osr4	311	75	60
Os_seed_1	chr01	4776238	4776546	LTR_fam51_osr4	308	68	37
Os_seed_1	chr04	21858386	21858681	LTR_fam51_osr4	295	59	14
Os_seed_1	chr04	21863682	21863990	LTR_fam51_osr4	308	59	58
Os_seed_1	chr08	25668346	25668726	LTR_fam51_osr4	380	55	41
Os_seed_1	chr04	21858330	21858527	LTR_fam51_osr4	197	50	15
Os_seed_1	chr10	22305984	22306180	LTR_fam51_osr4	196	48	26
Os_seed_1	chr08	14248353	14249088	LTR_fam4_dasheng_osr25	735	46	29
Os_seed_1	chr08	14248353	14249008	rire2_retrosat1	655	45	29
Os_seed_1	chr03	1916323	1916518	LTR_fam51_osr4	195	44	11
Os_seed_1	chr04	11804710	11805104	LTR_fam51_osr4	394	42	11
Os_seed_1	chr10	12426572	12427386	LTR_fam4_dasheng_osr25	814	35	10
Os_seed_1	chr10	19534197	19535685	LTR_fam33_osr30	1488	34	12
Os_seed_1	chr08	2557936	2559179	LTR_fam104	1243	31	12
Os_seed_1	chr08	25673742	25674136	LTR_fam51_osr4	394	28	11
Os_seed_1	chr09	8572142	8572329	LTR_fam51_osr4	187	27	29
Os_seed_1	chr03	16897683	16898505	LTR_fam4_dasheng_osr25	822	25	11
Os_seed_1	chr12	4390975	4391793	kangourou_osj	818	24	12
Os_seed_1	chr03	20764574	20764968	LTR_fam4_dasheng_osr25	394	24	14
Os_seed_1	chr06	12495693	12496498	LTR_fam4_dasheng_osr25	805	22	11
Os_seed_1	chr03	19223154	19223754	LTR_fam76	600	21	11
Os_seed_1	chr03	2173277	2173889	dagul	612	21	14
Os_seed_1	chr09	7815389	7816263	LTR_fam84	874	19	14
Os_seed_1	chr04	10330694	10331234	LTR_fam27_osr34	540	19	14
Os_seed_1	chr10	8866704	8867084	LTR_fam51_osr4	380	18	12
Os_seed_1	chr10	11146607	11147412	LTR_fam4_dasheng_osr25	805	16	14
Os_seed_1	chr06	14040040	14040843	LTR_fam4_dasheng_osr25	803	14	10
Os_seed_1	chr12	20399289	20400200	LTR_fam4_dasheng_osr25	911	14	10
Os_seed_1	chr03	19209738	19210021	LTR_fam40	283	13	10
Os_seed_1	chr09	1131747	1132066	rn_313-186	319	13	15
Os_seed_1	chr02	14853611	14854730	LTR_fam42	1119	12	14
Os_seed_1	chr05	14151923	14152468	LTR_fam33_osr30	545	11	11
Os_seed_1	chr08	18530632	18530848	LTR_fam51_osr4	216	10	11
Os_seed_2	chr02	11897228	11902686	LTR_fam51_osr4	5458	384	213
Os_seed_2	chr02	34010327	34015744	LTR_fam51_osr4	5417	365	101
Os_seed_2	chr04	31206043	31211501	LTR_fam51_osr4	5458	308	115
Os_seed_2	chr08	9051971	9057365	LTR_fam51_osr4	5394	291	27
Os_seed_2	chr01	4776416	4781875	LTR_fam51_osr4	5459	241	48
Os_seed_2	chr10	22300658	22306114	LTR_fam51_osr4	5456	197	102
Os_seed_2	chr09	8572210	8577669	LTR_fam51_osr4	5459	168	23
Os_seed_2	chr02	11897047	11897347	LTR_fam51_osr4	300	146	30
Os_seed_2	chr04	31211382	31211739	LTR_fam51_osr4	357	131	20
Os_seed_2	chr02	34010136	34010357	LTR_fam51_osr4	221	128	66
Os_seed_2	chr02	11897050	11897281	LTR_fam51_osr4	231	116	27
Os_seed_2	chr04	21863682	21863990	LTR_fam51_osr4	308	99	48
Os_seed_2	chr02	11902556	11902751	LTR_fam51_osr4	195	98	55
Os_seed_2	chr01	4776238	4776546	LTR_fam51_osr4	308	87	35
Os_seed_2	chr03	1911042	1915340	LTR_fam51_osr4	4298	85	11
Os_seed_2	chr02	34015614	34015809	LTR_fam51_osr4	195	80	13
Os_seed_2	chr04	21858386	21858681	LTR_fam51_osr4	295	67	14
Os_seed_2	chr10	22300480	22300791	LTR_fam51_osr4	311	60	43
Os_seed_2	chr03	1913340	1916453	LTR_fam51_osr4	3113	60	13
Os_seed_2	chr04	21858330	21858527	LTR_fam51_osr4	197	54	12
Os_seed_2	chr08	26495654	26495756	rn_408-218	102	47	43
Os_seed_2	chr08	26495662	26495797	rn_408-218	135	47	45
Os_seed_2	chr09	9834006	9834718	rn_128-73	712	44	12
Os_seed_2	chr04	31205978	31206173	LTR_fam51_osr4	195	43	41
Os_seed_2	chr10	22305984	22306180	LTR_fam51_osr4	196	40	24
Os_seed_2	chr07	26694787	26695362	LTR_fam158_tos17	575	38	12
Os_seed_2	chr09	17057819	17058433	dagul	614	36	11
Os_seed_2	chr12	16241280	16241646	LTR_fam67_echidne_osj	366	34	35
Os_seed_2	chr09	7648573	7648804	houba	231	31	17
Os_seed_2	chr08	7270463	7270600	rn_104-59	137	29	25

Os_seed_2	chr03	28646237	28646346	LTR_fam68	109	28	12
Os_seed_2	chr06	17910690	17911191	LTR_fam25	501	24	20
Os_seed_2	chr07	4779885	4780599	LTR_fam25	714	21	12
Os_seed_2	chr03	8228679	8228904	LTR_fam144	225	21	14
Os_seed_2	chr02	18177057	18177760	LTR_fam25	703	21	30
Os_seed_2	chr10	10222678	10223177	LTR_fam4_dasheng_osr25	499	20	14
Os_seed_2	chr01	22751593	22752283	LTR_fam25	690	17	20
Os_seed_2	chr11	8423653	8424158	hopi	505	16	17
Os_seed_2	chr07	12741240	12741367	rn_216-127	127	16	26
Os_seed_2	chr11	22408004	22408509	hopi	505	15	19
Os_seed_2	chr02	27694619	27695107	dendrobat_osj	488	15	11
Os_seed_2	chr08	25668346	25668726	LTR_fam51_osr4	380	14	11
Os_seed_2	chr05	12596949	12597677	LTR_fam42	728	11	16
